# Identification of Novel Aryl Carboxamide Derivatives as Death-Associated Protein Kinase 1 (DAPK1) Inhibitors with Anti-Proliferative Activities: Design, Synthesis, In Vitro, and In Silico Biological Studies

**DOI:** 10.3390/ph15091050

**Published:** 2022-08-25

**Authors:** Ahmed Elkamhawy, Sora Paik, Eslam M. H. Ali, Ahmed H. E. Hassan, So Jin Kang, Kyeong Lee, Eun Joo Roh

**Affiliations:** 1 BK21 FOUR Team and Integrated Research Institute for Drug Development, College of Pharmacy, Dongguk University-Seoul, Goyang 10326, Korea; 2 Department of Pharmaceutical Organic Chemistry, Faculty of Pharmacy, Mansoura University, Mansoura 35516, Egypt; 3 Chemical and Biological Integrative Research Center, Korea Institute of Science and Technology (KIST), Seoul 02792, Korea; 4 Department of Medicinal Chemistry and Molecular Pharmacology, Purdue University, 575 West Stadium Avenue, West Lafayette, IN 47907, USA; 5 Pharmaceutical Chemistry Department, Faculty of Pharmacy, Modern University for Technology and Information (MTI), Cairo 12055, Egypt; 6 Department of Medicinal Chemistry, Faculty of Pharmacy, Mansoura University, Mansoura 35516, Egypt; 7 Department of Biotechnology, Graduate School Korea University, Seoul 02841, Korea; 8 Division of Bio-Medical Science & Technology, University of Science and Technology, Daejeon 34113, Korea

**Keywords:** death-associated protein kinase 1 (DAPK1), kinase inhibitors, DAPK1 inhibitors, anti-proliferative activity, aryl carboxamides

## Abstract

Death-associated protein kinase 1 (DAPK1) is a serine/threonine protein kinase involved in diverse fundamental cellular processes such as apoptosis and autophagy. DAPK1 isoform plays an essential role as a tumor suppressor and inhibitor of metastasis. Consequently, DAPK1 became a promising target protein for developing new anti-cancer agents. In this work, we present the rational design and complete synthetic routes of a novel series of eighteen aryl carboxamide derivatives as potential DAPK1 inhibitors. Using a custom panel of forty-five kinases, a single dose of 10 µM of the picolinamide derivative **4a** was able to selectively inhibit DAPK1 kinase by 44.19%. Further investigations revealed the isonicotinamide derivative **4q** as a promising DAPK1 inhibitory lead compound with an IC_50_ value of 1.09 µM. In an in vitro anticancer activity assay using a library of 60 cancer cell lines including blood, lung, colon, CNS, skin, ovary, renal, prostate, and breast cancers, four compounds (**4d**, **4e**, **4o**, and **4p**) demonstrated high anti-proliferative activity with mean % GI ~70%. Furthermore, the most potent DAPK1 inhibitor (**4q**) exhibited remarkable activity against leukemia (K-562) and breast cancer (MDA-MB-468) with % GI of 72% and 75%, respectively.

## 1. Introduction

Death-associated protein kinase 1 (DAPK1) (a Ca^2+^/calmodulin dependent Ser/Thr kinase) is an essential mediator in cell death and autophagy related signals [1,2]. It consists of 1430 residues and is the largest member in the DAPK protein family, including a Ca^2+^/CaM autoregulatory domain, a death domain, and a serine-rich C-terminal tail whose phosphorylation activity is known to be responsible for specific forms of apoptosis [3,4]. It coordinates cell-death signaling pathways in response to various stimuli such as death receptor activation, cytokines, matrix detachment, ceramide, ischemia, and glutamate toxicity [5]. As previously discussed in various studies, DAPK1 as a stress-responsive kinase is a crucial component that transmits ER stress signals into two distinct directions, caspase activation (via regulating type I apoptotic caspase-dependent cell death) and autophagy (by controlling type II autophagic caspase-independent cell death) [5,6,7,8,9,10,11,12,13].

**Figure 1:**
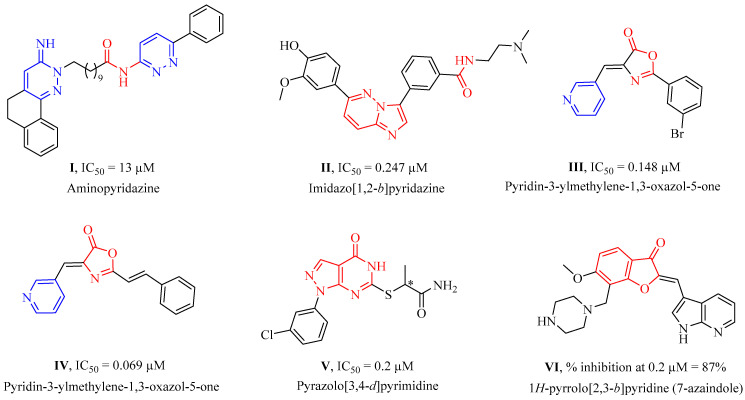
Reported synthetic small molecules bearing different chemical scaffolds and their IC_50_ values or % inhibition against DAPK1 kinase.

Among novel effective approaches to hinder apoptosis pathways, some fusion proteins have been reported [14]. However, a peptide-based strategy has certain potential scientific and technical cautions, such as lack of cell selectivity, instability, as well as uncertainty of the effective therapeutic concentration, which influences the peptide cargo in addition to suffering from rapid degradation after administration orally [15]. Hence, the rational drug design of small molecule inhibitors could be a unique way to overcome such drawbacks. Although DAPK1 has gained a lot of interest regarding the comprehension of its functions, only a small number of chemical scaffolds, comprehensively discussed in our recent review [4], have been found in the literature with DAPK1 inhibitory activity, i.e., aminopyridazine [16,17], imidazo [1,2-*b*]pyridazine [18], pyridin-3-ylmethylene-1,3-oxazol-5-one [19,20], pyrazolo [3,4-*d*]pyrimidinone [21,22], and 1*H*-pyrrolo [2,3-*b*]pyridine (7-azaindole) [23] (Figure 1). Inspired by the various unsolved issues of reported scaffolds, such as instability in biological systems, low potency, low selectivity profile, and/or insufficient toxicity studies, in addition to the absence of a current promising clinical candidate or an FDA-approved specific DAPK1 inhibitor, our institute has launched a project aimed at designing novel leads for DAPK1 activity modulation with potential anticancer activities.

In previous studies, a structure-based virtual screening strategy was followed to develop an oxazol-5-one derivative **III** and its related analogues as DAPK inhibitors. Further in silico studies were conducted to define the structure–activity relationship (SAR) of the developed inhibitors [19,24]. The SAR studies stated that a nitrogen containing group, such as pyridinyl moiety, is essential for DAPK activity due to the role of the N-atom in H-bond formation with the backbone NH of Val96 in the hinge region at the ATP binding site. In addition, substitution on the terminal phenyl ring was found to improve potency against DAPK compared to the unsubstituted analogues, which might be explained by the increase in electron density of the phenyl ring which allows for ring contribution in hydrophobic interactions at the ATP binding site. Regarding phenyl ring substitution, the studies reported that meta-substitution seems to be more appropriate for binding at the ATP binding site than para-substitution because of electronic effects. Additionally, substitution with electron withdrawing groups at meta-positions is more effective for activity than electron donating ones. It was also observed that the phenyl ring is located near Asp161 in a very tight area of the binding pocket, so bulky substitution would badly affect inhibitor affinity to the binding site and reduce their stability and activity, as well. Finally, docking of compound **III** in DAPK binding sites illustrated an observed vacancy around the phenyl group, which is considered a point of optimization for developing new DAPK inhibitors with extended substitution on the phenyl ring. 

**Figure 2:**
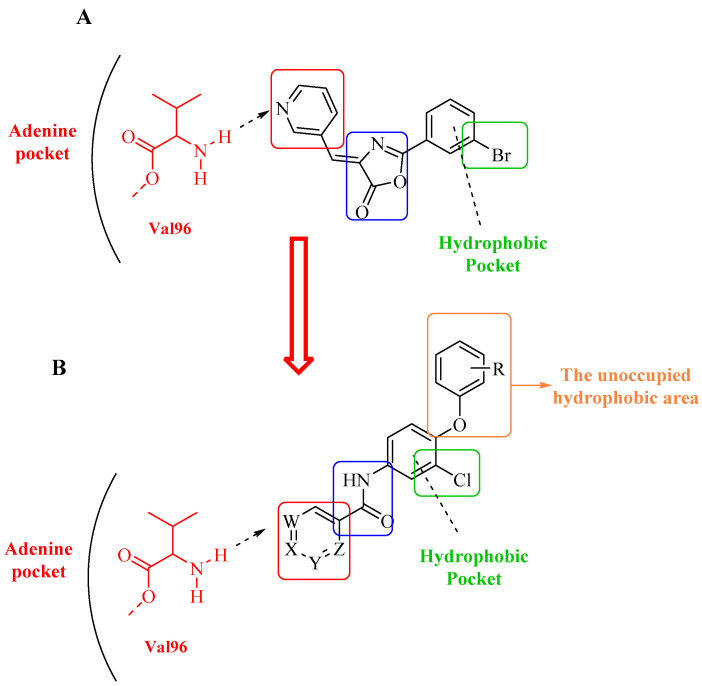
Diagrammatic representation of the structural modifications of compound **III** for developing new DAPK inhibitor candidates: (**A**) molecular interactions of compound **III** into the DAPK active site; (**B**) proposed binding interaction of the new synthesized derivatives **4a**–**r** into the DAPK kinase domain (W, X, Y, and Z could be C or N atoms).

Relying on the aforementioned SAR studies, structural optimizations of the reported inhibitor **III** were conducted, while keeping the essential binding interactions, with the aim of producing a novel series of DAPK inhibitors (**4a**–**r**) (Figure 2). In the current work, the designed carboxamide derivatives (**4a**–**r**) were designed to retain the hinge binding interaction with the Val96 backbone by introducing different nitrogen containing groups (pyridine, pyridazine, and pyrazine). However, the principal modification was the ring opening of the central oxazole into carboxamide moiety, which has been reported in many DAPK inhibitors. The lateral phenyl group of the designed series was substituted by an electron withdrawing meta-chloro group that was reported for better hydrophobic interaction. The new designed compounds possessed a unique extension in the hydrophobic area via additional phenoxy substitutions on the terminal phenyl group in an attempt to boost molecular interaction with the enzyme pocket.

## 2. Results and Discussion

### 2.1. Chemistry

**Scheme 1:**
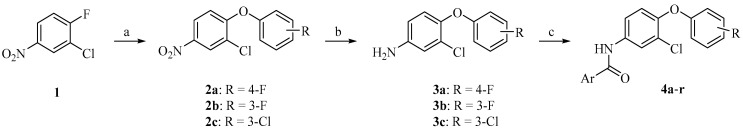
Reagents and conditions: (**a**) appropriate phenol derivative, K_2_CO_3_, MeCN, 85 °C, 6 h; (**b**) H_2_, Pt/C, MeOH, rt, 6 h; (**c**) appropriate benzoic acid derivative, DIPEA, HATU, THF, reflux, overnight.

The chemical synthesis of the target compounds **4a**–**r** was carried out as illustrated in Scheme 1. A nucleophilic aromatic substitution reaction of the commercially available starting material 2-chloro-1-fluoro-4-nitrobenzene (**1**) was performed via adding the appropriate phenolic derivative and a catalytic amount of potassium carbonate to generate 2-chloro-4-nitrophenoxybenzene derivatives (**2a**–**c**). Catalytic hydrogenation of compounds **2a**–**c** was performed using Pt/C catalyst under hydrogen atmosphere to yield compounds **3a**–**c**. Introduction of amide functionality to intermediate **3a**–**c** was achieved by coupling with the appropriate benzoic acid derivative using HATU to afford the final target compounds **4a**–**r** (Table 1).

### 2.2. Biological Evaluation

#### 2.2.1. Evaluation of Compound **4a** against a Panel of 45 Kinases

In an attempt to test our design hypothesis and discover if DAPK1 is the potential conceivable kinase target of the designed candidates, a kinase selectivity assay was carried out to the first synthesized derivative (**4a**) against a group of forty-five different kinases. Compound **4a** was evaluated at a single dose concentration of 10 μM and % inhibition was determined against each corresponding kinase (Table 2).

**Figure 3:**
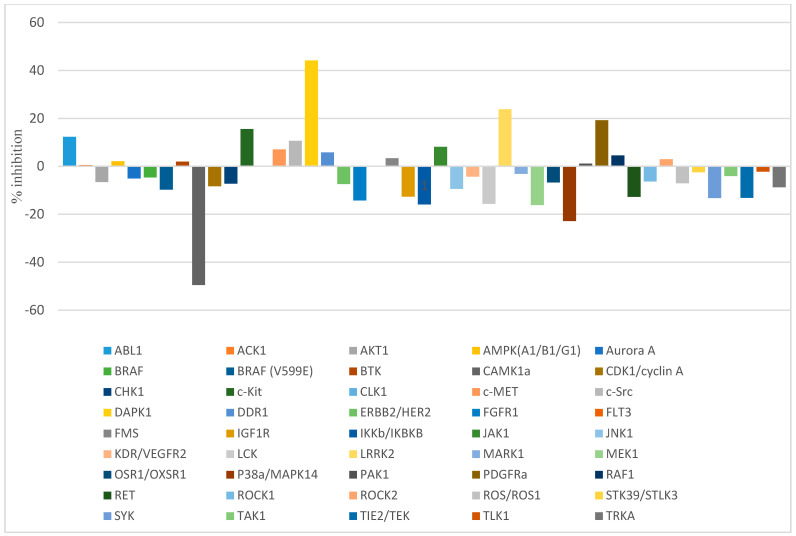
A schematic chart illustrating the enzyme inhibitory activity of compound **4a** (10 µM) against a panel of 45 oncogenic kinases.

Interestingly, the preliminary data revealed a remarkable selectivity of compound **4a** towards DAPK1, with mean % inhibition of 44.19%. On the other hand, the results for the other 44 kinases showed no activity for most of the kinases and low activity (less than 25%) for a few of the tested kinases (Figure 3). An additional inspection of compound **4a** selectivity was carried out by evaluating its activity against other DAPK isoforms (DAPK2 and DAPK3). The obtained results showed no activity for compound **4a** with DAPK2 and DAPK3 isoforms (data not shown).

#### 2.2.2. In Vitro DAPK1 Kinase Assay and Optimization towards Lead Development

Based on the previous panel of kinase data, a series of aryl carboxamide derivatives (**4b**–**r**) were synthesized and evaluated in vitro for their inhibitory activity against DAPK1 using the ELISA technique (enzyme-linked immune sorbent assay). The enzyme inhibition assay was conducted with a 10 μM dose of the tested compounds and the mean % inhibition was measured. As illustrated in Table 3, derivatives possessing the pyridinyl carboxamide moiety (**4c**, **4e**, **4f**, **4h**, **4j**, **4k**, **4m**, **4p**, and **4q**) exhibited the highest inhibitory activity (59–81%) in comparison to the pyridazine or pyrazine possessing derivatives (38–61%). In addition, the attachment point of these nitrogen heterocycles (pyridine, pyridazine, and pyrazine) with the amide group did not show a remarkable effect in terms of activity. In regard to the substitution on the terminal phenoxy moiety, the overall results showed that para-substitution with the fluoro group in compounds **4a** and **4h**–**l** has a better effect on activity than the meta-substituted derivatives **4b**–**g**, except for the pyridazine bearing derivatives **4a** and **4b**. Moreover, replacing the m-fluoro substitution with *m*-chloro in compounds **4m**–**r** showed a slight improvement in activity, except in compounds **4d** and **4o** where the chloro substitution dramatically reduced activity to 37.99%. Among the tested series, compounds **4h**, **4j**, **4k,** and **4q** revealed the highest activity of 81%, 79%, 80%, and 72%, respectively.

#### 2.2.3. Dose-Dependent Assay of Compounds **4h**, **4j**, **4k**, and **4q** with DAPK1 Kinase

For more clarification of the structural activity relationship of the synthesized compounds, the most active derivatives (**4h**, **4j**, **4k** ,and **4q)** were subjected to a dose-dependent IC_50_ kinase assay. The selected candidates showed low micromolar IC_50_ values against DAPK1 kinase with a range of 1.09–7.26 μM. The data obtained in Table 4 revealed the impact of the attachment point of the pyridine moiety with the amide linker and, for the 4-F phenoxy derivatives, nicotinamide owing compound 4j exhibited the highest activity (IC_50_ = 1.7 μM). In contrast, substitution with picolinamide (**4h**) and isonicotinamide (**4k**) declined activity to 6.81 µM and 7.26 µM, respectively. Interestingly, the isonicotinamide derivative with the terminal *m*-chlorophenoxy moiety (**4q**) emerged to be the most potent inhibitor with an IC_50_ value of 1.09 µM. In order to determine its selectivity against DAPK1, compound **4q** was evaluated at a single dose concentration (10 µM) against the other two isoforms (DAPK2/DAPK3). The two isoforms exhibited no inhibition at all by compound **4q** (data not shown), indicating the high potential of compound **4q** to be a promising selective DAPK1 inhibitory lead compound.

#### 2.2.4. In Vitro Anticancer Assay

**Figure 4:**
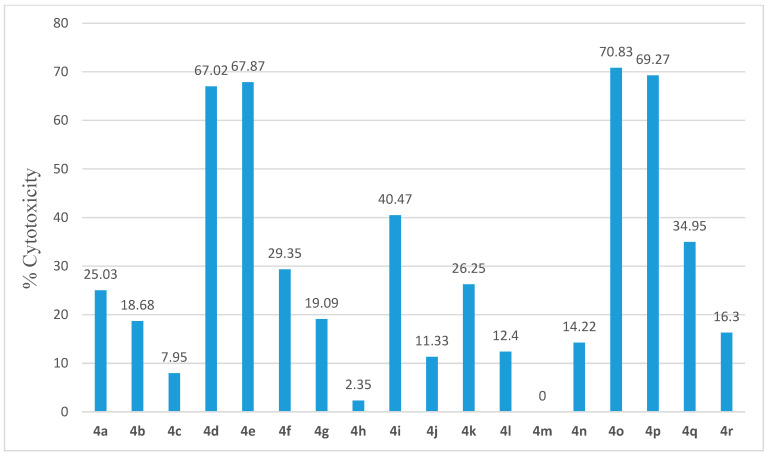
Mean % inhibition of NCI-60 cell lines after treatment with a single dose (10 µM) of compounds **4a**–**r**.

The anticancer effects of all newly synthesized compounds (**4a**–**r**) were assessed through determining their anti-proliferative activities against a panel of NCI-60 human cancer cell lines belonging to nine cancer types (leukemia, non-small cell lung cancer, colon cancer, CNS cancer, melanoma, ovarian cancer, renal cancer, prostate cancer, and breast cancer). All the subjected compounds were selected for one-dose 60 cell line assays. The tested compounds showed wide spectrum activity against the different human cancer cell lines with maximum mean inhibition of 71% (Figure 4). Among the tested compounds, only compounds **4d**, **4e**, **4o**, and **4p** displayed high anti-proliferative activities (67.02%, 67.87%, 70.83%, and 69.27%, respectively). The data obtained from NCI assays are analyzed in this section.

**Figure 5:**
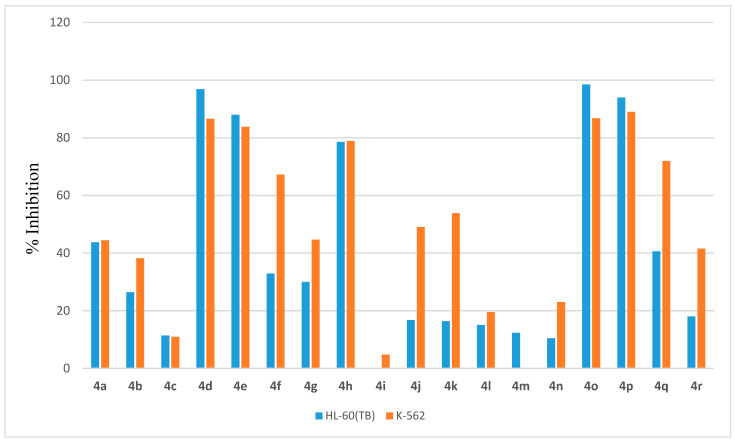
Mean % inhibition of the target compounds **4a**–**r** against leukemia cell lines (HL-60(TB) and K-562).

■ Anti-proliferative activity against leukemia:

The efficacy of the synthesized compounds against leukemia cell lines (CCRF-CEM, HL-60(TB), K-562, MOLT-4, RPMI-8226, and SR) is illustrated in Table 5. It was observed that leukemia cells (HL-60(TB) and K-562) are the most sensitive cell lines towards treatment with the tested compounds (Figure 5). The pyrazine carboxamide derivatives **4d** and **4o**, with meta-(F or Cl) substitution on the terminal phenoxy group, totally inhibited the growth of the leukemia HL-60(TB) cell line with almost 100% inhibition and showed above 90% growth inhibition for leukemia K-562. Compounds **4e**, **4h**, and **4p** exhibited satisfactory inhibitory activity against both cell lines (78–83%), while most of the tested candidates showed moderate anti-proliferative activity against leukemia (30–60%).

■ Anti-proliferative activity on non-small cell lung cancer:

The synthesized candidates were evaluated against a panel of nine NSCLCs (A549, EKVX, HOP62, HOP92, NCI-H226, NCI-H23, NCI-H322M, NCI-H460, and NCI-H522). The results are represented in Table 6. Among the tested series, compounds **4d**, **4e**, **4o**, and **4p** were the most active anti-proliferative agents against the nine NSCLCs (% GI = 55–60%).

**Figure 6:**
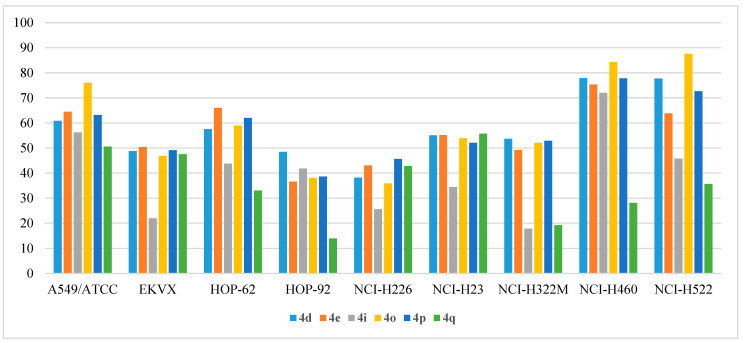
Anti-proliferative activities (% GI) of compounds **4d**, **4e**, **4i**, **4o**, **4p**, and **4q** against NSCLCs.

As depicted in Figure 6, NCI-H460 and NCI-H522 were the highest affected cell lines by the mentioned active compounds. NCI-H460 showed % GI of 77.9%, 75.34%, 84.28%, and 77.8%, while NCI- H522 growth was inhibited by 77.7%, 63.79%, 87.25%, and 72.65% upon treatment with 10 µM of **4d**, **4e**, **4o**, and **4p**, respectively. Compounds **4i** and **4q** elicited moderate activity with average inhibition of 40% and 36%, respectively. Meanwhile, the rest of the tested series did not exhibit significant activity towards NSCLCs (average % GI < 30%).

**Figure 7:**
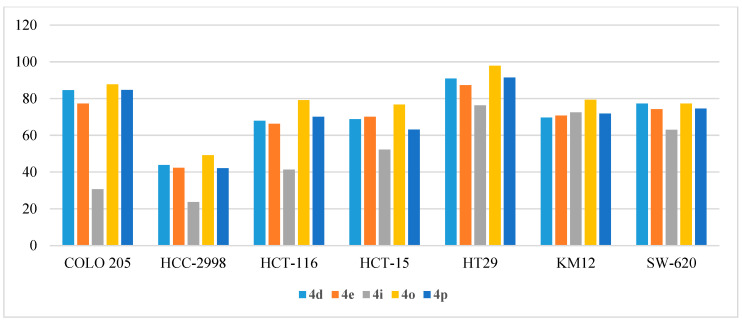
Anti-proliferative activities (% GI) of compounds **4d**, **4e**, **4i**, **4o**, and **4p** against colon cancer cell lines.

■ Anti-proliferative activity on colon cancers:

The target compounds were assayed against a panel of seven colon adenocarcinoma cell lines (COLO-205, HCC-2998, HCT-116, HCT-15, HT29, KM12, and SW-620) and the results are summarized in Table 7. The tested compounds revealed a wide range of inhibitory activity against the seven cell lines, with average % GI from 0% to 78%. The overall data indicated that compounds **4d**, **4e**, **4o**, and **4p** had the highest growth inhibitory activity and showed average % GI of 71.84%, 69.73%, 78.16%, and 71.11%, respectively (Figure 7). Compounds **4d** and **4e** significantly inhibited the growth of HOP92 adenocarcinoma with % GI of 90.87% and 87.13%, respectively. The two compounds also exhibited outstanding activity towards the COLO 205 cell line (% GI = 84.52% and 77.29%, respectively).

Moreover, compounds **4o** and **4p** emerged to be the most potent inhibitors of HOP92 adenocarcinoma with % GI of 97.8% and 91.4%, respectively. Furthermore, the COLO 205 cell line was also inhibited by the two compounds with % GI of 87.71% and 84.61% for the two compounds. Compound **4i** moderately inhibited the growth of HCT-15, HT29, KM12, and SW-620 and exhibited % GI of 52.26%, 76.34%, 72.49%, and 63.04%, respectively (Figure 7).

**Figure 8:**
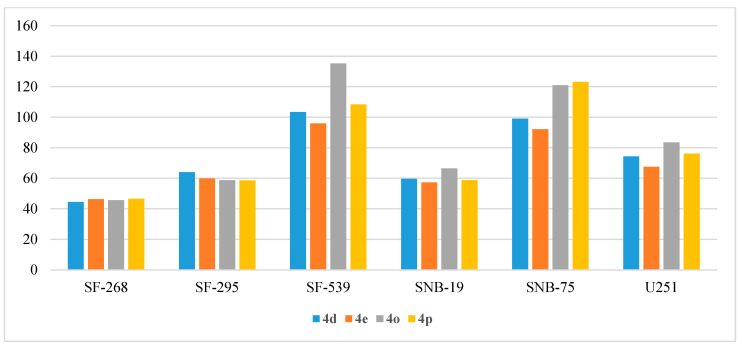
Anti-proliferative activities (% GI) of compounds **4d**, **4e**, **4o**, and **4p** against CNS cancer cell lines.

■ Anti-proliferative activity on CNS cancers:

The anti-proliferative activities of the target derivatives were determined against six CNS cancer cell lines (SF-268, SF-295, SF-539, SNB-19, SNB-75, and U251) and the resulting % GIs were tabulated in Table 8. As illustrated, compounds **4d**, **4e**, **4o**, and **4p** kept their ranking among the tested derivatives as the most potent anti-proliferative agents. It was observed that SF-539 and SNB-75 were the most sensitive cell lines that showed growth inhibition around 100% upon treatment with 10 µM of compounds **4d**, **4o**, **4p**, **4e** (Figure 8). In addition, the mentioned derivatives broadly inhibited the growth of the remaining four cell lines (44.50–83.53%). Compound **4q** moderately inhibited the growth of SF-295 (67.42%), while there was no observable activity from the compound towards the other five cell lines.

**Figure 9:**
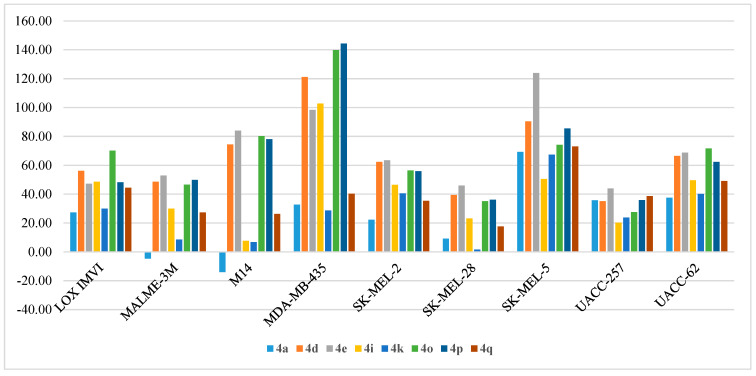
Anti-proliferative activities (% GI) of compounds **4a**, **4d**, **4e**, **4i**, **4k**, **4o**, **4p**, and **4q** against Melanoma cell lines.

■ Anti-proliferative activity on melanoma:

Melanoma anti-proliferative activity of the synthesized compounds was evaluated using a panel of nine melanoma cell lines (LOX IMVI, MALME-3M, M14, MDA-MB-435, SK-MEL-2, SK-MEL-28, SK-MEL-5, UACC-257, and UACC-62) with the % GI results illustrated in Table 9. The highest potency was observed on the MDA-MB-435 cell line which was strongly inhibited by compounds **4d**, **4e**, **4i**, **4o**, and **4p** (% GI = 121%, 98%, 102%, 139%, and 144%, respectively) (Figure 9). Compound **4e** exhibited high potency against the SK-MEL-5 cell line with % GI of 124%, while the same cell line was highly inhibited by **4d**, **4o**, **4p**, and **4q** (% GI of 90.39%, 74.19%, 85.47%, and 73.04%, respectively) and moderately inhibited by **4a**, **4f**, **4i**, and **4k** (% GI of 69.25%, 58.84%, 50.48%, and 67.41%, respectively). Moreover, compounds **4o** and **4p** showed moderate activity against the M14 cell line (80.21% and 78.15%, respectively) and UACC-62 cell line (71.64% and 62.37%, respectively).

**Figure 10:**
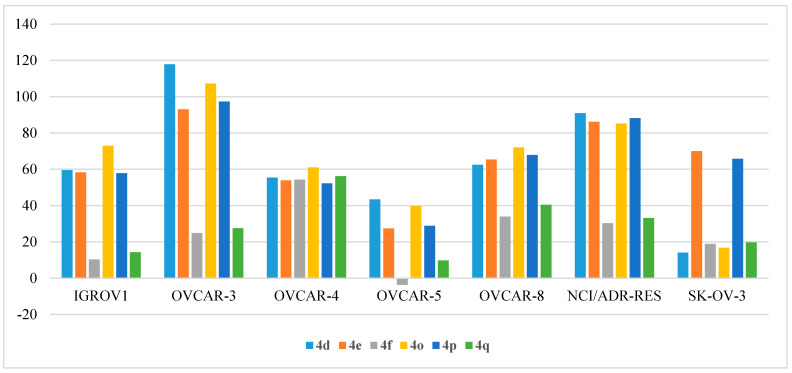
Anti-proliferative activities (% GI) of compounds **4d**, **4e**, **4f**, **4o**, **4p**, and **4q** against ovarian cancer cell lines.

■ Anti-proliferative activity on ovarian cancers:

The growth inhibition results of the tested compounds against a panel of six ovarian cancer cell lines are demonstrated in Table 10. Among the employed ovarian cell lines, OVCAR-3 emerged to be the most sensitive cell line, which was strongly inhibited by compounds **4d**, **4e**, **4o**, and **4p** with % GI of 117.85%, 93.03%, 107.23%, and 97.34%, respectively. The **4d**, **4e**, **4o**, and **4p** compounds also inhibited the growth of the NCI/ADR-RES cell line with % GI over 85%. OVCAR-3 and NCI/ADR-RES were moderately inhibited by compound **4i** (56.36% and 59.16%, respectively). In addition, moderate growth inhibition activity was detected against three cell lines: SK-OV-3 by compounds **4e** and **4p** (69.98% and 65.75%, respectively); OVCAR-8 by compounds **4d**, **4e**, **4o**, and **4p** (62.49%, 65.37%, 72.05%, and 67.92%, respectively); OVCAR-4 by compounds **4d**, **4e**, **4f**, **4o**, **4p,** and **4q** (55.46%, 53.83%, 54.30%, 61.05%, 52.16%, 56.18%, respectively); and IGROV1 by compounds **4d**, **4e**, **4o**, and **4p** (59.49%, 58.28%, 72.96%, 57.85%, respectively) (Figure 10).

**Figure 11:**
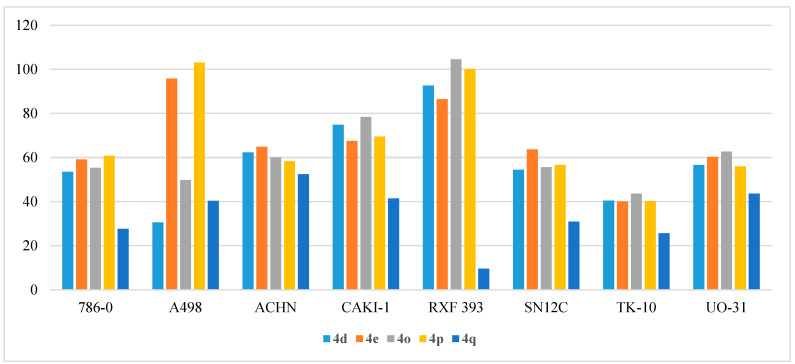
Anti-proliferative activities (% GI) of compounds **4d**, **4e**, **4o**, **4p**, and **4q** against renal cancer cell lines.

■ Anti-proliferative activity on renal cancers:

The synthesized compounds **4a**–**r** exhibited a wide range of inhibitive activity towards a panel of eight renal cancer cell lines (786-0, A498, ACHN, CAKI-1, RXF 393, SN12C, TK-10, and UO-31), as shown in Table 11. The uppermost activity was observed against the growth of the RXF 393 cell line which was totally inhibited (% GI > 100%) by compounds **4o** and **4p** and greatly inhibited by compounds **4d** and **4e** (92.63% and 86.46%, respectively). Additionally, the A498 cell line showed inhibition of 100% by compound **4p** and 95.84% by compound **4e** (Figure 11). Modest activity was noticed against the 786-0 cell line by compounds **4d**, **4e**, **4o**, and **4p** (% GI of 53.47%, 59.13%, 55.36%, and 60.79%, respectively), the ACHN cell line by compounds **4d**, **4e**, **4o**, **4p**, and **4q** (% GI of 62.30%, 64.82%, 60.04%, 58.34%, and 52.45%, respectively), the CAKI-1 cell line by compounds **4d**, **4e**, **4o**, and **4p** (% GI of 74.84%, 67.45%, 78.37%, and 69.44%, respectively), the SN12C cell line by compounds **4d**, **4e**, **4o**, and **4p** (% GI of 54.42%, 63.63%, 55.60%, and 56.56%, respectively), and the UO-31 cell line by compounds **4d**, **4e**, **4o**, and **4p** (% GI of 56.60%, 60.28%, 62.66%, and 55.92%, respectively) (Figure 11).

**Figure 12:**
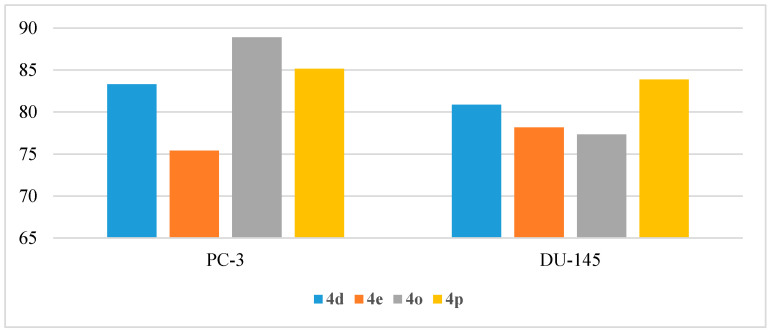
Anti-proliferative activities (% GI) of compounds **4d**, **4e**, **4o**, and **4p** against prostate cancer cell lines.

■ Anti-proliferative activity on prostate cancers:

Two prostate cancer cell lines (PC-3 and DU-145) were used for the anti-proliferative evaluation of the target compounds **4a**–**r** (Table 12). Among the tested series, only compounds **4d**, **4e**, **4o**, and **4p** exhibited satisfactory potency against the two cell lines. The four candidates inhibited the growth of the PC-3 cell line by % GI of 83.31%, 75.41%, 88.90%, and 85.15%, respectively. However, the DU-145 cell line was inhibited by % GI of 80.87%, 78.17%, 77.35%, and 83.88% by the four compounds, respectively (Figure 12).

**Figure 13:**
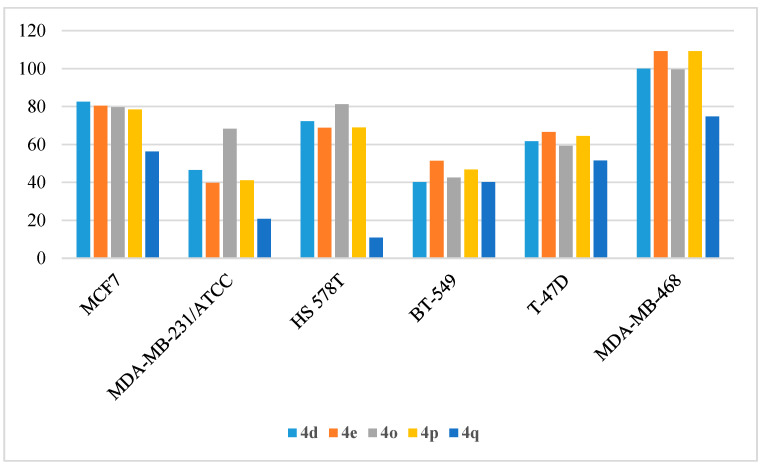
Anti-proliferative activities (% GI) of compounds **4d**, **4e**, **4o**, **4p**, and **4q** against breast cancer cell lines.

■ Anti-proliferative activity on breast cancers:

Finally, the target compounds were evaluated as anti-proliferative agents against six different breast cancer cell lines (MCF7, MDA-MB-231/ATCC, HS 578T, BT-549, T-47D, and MDA-MB-468) and the results were summarized. As shown in Table 13, the growth of the MDA-MB-468 cell line was completely inhibited (100–109%) by compounds **4d**, **4e**, **4o**, and **4p**, while its growth was moderately inhibited by compounds **4a**, **4f**, **4g**, **4i**, **4k**, **4q**, and **4r** (52–76%). The MCF7 cell line was less sensitive to the tested compounds and showed maximum growth inhibition of 82.54% and 80.44% with compounds **4d** and **4e**, respectively. The HS 578T and T-47D cell lines were moderately inhibited by compounds **4a**, **4d**, **4e**, **4o**, and **4p** (% GI 59.27–81.27%). MDA-MB-231/ATCC and BT-549 were the most resistant cell lines to the tested compounds, except for compound **4o** that inhibited the growth of MDA-MB-231/ATCC by % GI of 68.29% (Figure 13).

#### 2.2.5. Docking Study

**Figure 14:**
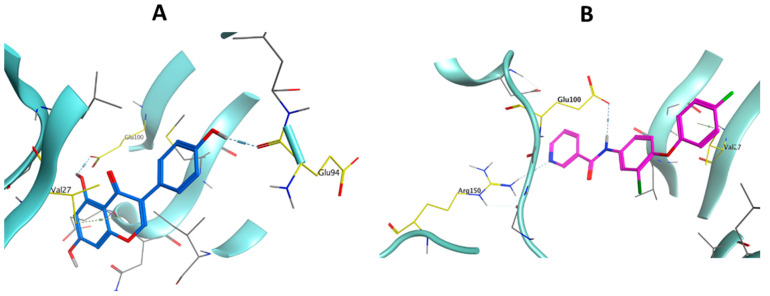
3D representation of Genistein (**A**) and compound **4j** (**B**) molecular interactions in the DAPK1 kinase.

In an attempt to introduce a reasonable explanation for the observed DAPK1 kinase activity with respect to the inhibitors’ binding affinity, the potent inhibitor **4j** was subjected to a molecular docking study as a representative example of the designed inhibitors. In this study, Molecular Operating Environment (MOE, 2014) software was used to operate the docking protocol. The X-ray crystallographic structures of DAPK1 in complex with Genistein (PDB ID: 5AUZ) were downloaded from the protein data bank (PDB). Validation of the docking protocol was achieved by re-docking of the co-crystalized ligand, Genistein, in the binding site of DAPK1. The re-docked ligand retained the same binding manner in the DAPK1 active site (docking score= −6.1511 kcal/mol) with an RMSD value of 0.8894 Å. As illustrated in Figure 14A, the iso-flavone moiety of the native ligand, Genistein, is binding in the adenine pocket via H-bonding between the hydroxyl group and Glu100 residue while the pyran ring is additionally embedded in the pocket by AreneH interaction with Val27. Furthermore, the DAPK1 hydrophobic pocket is occupied by the lateral hydroxy phenyl moiety, which exhibits H-bonding between its para-hydroxyl group and the Glu94 residue. Accordingly, the target compounds should conserve the binding mode of the native ligand, which in turn would also be an indication of their binding affinity and enzymatic activity. In Figure 14B, docking of compound **4j** in the active site of DAPK1 (docking score= -5.5545 kcal/mol) illustrates that the NH of the amide linker is introduced to the adenine pocket and forms H-bonding with Glu100, while the phenoxy moiety is inserted deeply in the pocket via Arene-H interaction with Val27. On the other hand, the pyridine ring, which is substituted on the amide linker, exhibits additional H-bonding with Arg150.

## 3. Materials and Methods

### 3.1. Chemistry

General: All reactions and manipulations were performed in a nitrogen atmosphere using standard Schlenk techniques. All reaction solvents and reagents were purchased from commercial suppliers and used without further purification. The NMR spectra were obtained with a Bruker Avance 400 (400 MHz ^1^H and 100.6 MHz ^13^C NMR). ^1^H NMR spectra were referenced to tetramethylsilane (δ = 0.00 ppm) as an internal standard and were reported as follows: chemical shift, multiplicity (b = broad, s = singlet, d = doublet, t = triplet, dd = doublet of doublet, m = multiplet). Column chromatography was performed on Merck Silica Gel 60 (230–400 mesh) and eluting solvents for all of these chromatographic methods were noted as appropriated-mixed solvent with given volume-to-volume ratios. TLC was carried out using glass sheets pre-coated with silica gel 60 F_254_ purchased by Merk. For more details, see Supplementary Files

#### 3.1.1. General Procedure of 2-Chloro-4-nitrophenoxybenzene Derivatives (**2a**–**c**)

Potassium carbonate (1.25 mmol) and the appropriate phenol (1 mmol) were added to a solution of commercially available 2-chloro-1-fluoro-4-nitrobenzene (1 mmol) in MeCN (12 mL). The reaction mixture was heated at 85 °C for 6 h. The mixture was extracted with EtOAc and water. The organic layer was dried over Na_2_SO_4_ and concentrated under reduced pressure to give the target compound.

2-Chloro-1-(4-fluorophenoxy)-4-nitrobenzene (**2a**)

Yellowish white solid, yield: 77%, mp: 76.3−92.4 °C, ^1^H NMR (400 MHz, CDCl_3_) δ 6.76 (d, *J* = 8.9 Hz, 1H), 7.00−7.09 (m, 4H), 7.98 (d, *J* = 8.9 Hz, 1H), 8.31 (s, 1H) [25,26].

2-Chloro-1-(3-fluorophenoxy)-4-nitrobenzene (**2b**)

Yellow oil, yield: 96%, ^1^H NMR (400 MHz, CDCl_3_) δ 6.80−6.87 (m, 2H), 6.95−7.00 (m, 2H), 7.40 (dd, *J* = 8.2 Hz, 14.6 Hz, 1H), 8.10 (dd, *J* = 2.7 Hz, 9.0 Hz, 1H), 8.40 (d, *J* = 2.7 Hz, 1H). ^13^C NMR (100.6 MHz, CDCl_3_) δ 107.68 (*J*_C−F_ = 24.1 Hz), 112.49 (*J*_C−F_ = 21.1 Hz), 115.27 (*J*_C−F_ = 3.0 Hz), 117.91, 123.69, 125.39, 126.66, 131.27 (*J*_C−F_ = 9.1 Hz), 143.29, 115.83 (*J*_C−F_ = 10.1 Hz), 158.07, 163.58 (*J*_C−F_ = 248.5 Hz) [27].

2-Chloro-1-(3-chlorophenoxy)-4-nitrobenzene (**2c**)

Yellow solid, yield: 90%, mp: 92.4−93.1 °C, ^1^H NMR (400 MHz, CDCl_3_) δ 6.95−6.98 (m, 2H), 7.09 (s, 1H), 7.24 (d, *J* = 8.0 Hz, 1H), 7.37 (t, *J* = 8.1 Hz, 1H), 8.09 (dd, *J* = 2.0 Hz, 9.0 Hz, 1H), 8.38 (d, *J* = 2.0 Hz, 1H). ^13^C NMR (100.6 MHz, CDCl_3_) δ 117.84, 117.91, 120.22, 123.71, 125.36, 125.71, 126.67, 131.15, 135.73, 143.28, 155.41, 158.07 [28].

#### 3.1.2. General Procedure of 3-Choro-4-phenoxyaniline Derivatives (**3a**–**c**)

Pt/C catalyst was added to a solution of the appropriate 2-chloro-4-nitrophenoxybenzene derivative (0.1 mmol) in MeOH (15 mL) under hydrogen atmosphere. The mixture was stirred at room temperature for 6 h. The reaction mixture was filtered with celite and washed with MeOH. The filtrate was evaporated in vacuo. The residue was purified by column chromatography to give the target compound.

3-Chloro-4-(4-fluorophenoxy) aniline (**3a**)

Brown oil, yield: 65%, ^1^H NMR (400 MHz, DMSO-*d_6_*) δ 5.34 (s, 2H), 6.55 (d, *J* = 8.8 Hz, 1H), 6.72 (s, 1H), 6.80–6.84 (m, 2H), 6.90 (d, *J* = 8.6 Hz, 1H), 7.13 (t, *J* = 8.2 Hz, 2H) [29].

3-Chloro-4-(3-fluorophenoxy) aniline (**3b**)

Yellow oil, yield: 78%, ^1^H NMR (400 MHz, DMSO-*d_6_*) δ 5.40 (s, 2H), 6.57 (d, *J* = 8.6 Hz, 1H), 6.63 (d, *J* = 8.4 Hz, 2H), 6.73 (s, 1H), 6.86 (t, *J* = 8.5 Hz, 1H), 6.96 (d, *J* = 8.8 Hz, 1H), 7.33 (t, *J* = 8.0 Hz, 1H) [27,30].

3-Chloro-4-(3-chlorophenoxy) aniline (**3c**)

Brown oil, yield: 60%, ^1^H NMR (400 MHz, DMSO-*d_6_*) δ 5.41 (s, 2H), 6.58 (dd, *J* = 2.6 Hz, 8.7 Hz, 1H), 6.73 (d, *J* = 2.6 Hz, 1H), 6.77–6.82 (m, 2H), 6.96 (d, *J* = 8.6 Hz, 2H), 7.09 (dd, *J* = 1.0 Hz, 8.0 Hz, 1H), 7.34 (t, *J* = 8.1 Hz, 1H) [28,30].

#### 3.1.3. General Procedure of Target Compounds **4a**–**r**

The appropriate 3-chloro-4-phenoxyaniline derivative (0.1 mmol) was dissolved in THF (12 mL), followed by adding DIPEA (0.27 mmol), HATU (0.11 mmol), and the appropriate benzoic acid derivative (0.1 mmol). The reaction mixture was refluxed overnight. The mixture was extracted with EtOAc, water, and brine. The organic layer was dried over Na_2_SO_4_ and concentrated under reduced pressure. The residue was purified by column chromatography to give the target compound.

*N*-(3-chloro-4-(4-fluorophenoxy) phenyl) picolinamide (**4a**)

Brown solid, mp: 95.6–97.0 °C, ^1^H NMR (400 MHz, CDCl_3_) δ 6.91–6.95 (m, 2H), 7.01 (dd, *J* = 8.8 Hz, 17.2 Hz, 3H), 7.49–7.52 (m, 1H), 7.60 (dd, *J* = 2.4 Hz, 8.8 Hz, 1H), 7.92 (t, *J* = 7.6 Hz, 1H), 8.03 (d, *J* = 2.4 Hz, 1H), 8.29 (d, *J* = 7.8 Hz, 1H), 8.61 (d, *J* = 4.6 Hz, 1H), 10.04 (s, 1H). ^13^C NMR (100.6 MHz, CDCl_3_) δ 116.30 (*J*_C−F_ = 23.1 Hz), 118.91, 119.16 (*J*_C−F_ = 31.2 Hz), 121.03, 121.97, 122.48, 126.22, 126.72, 134.55, 137.82, 148.05, 148.89, 149.37, 153.14, 158.69 (*J*_C−F_ = 241.4 Hz), 162.02. HRMS (ESI) *m/z* calculated for C_17_H_11_ClFN_3_NaO_2_ [M+Na]^+^: 366.0416, found: 366.0408.

*N*-(3-chloro-4-(4-fluorophenoxy) phenyl) pyridazine-3-carboxamide (**4b**)

White solid, mp: 134.1–134.9 °C, ^1^H NMR (400 MHz, CDCl_3_) δ 6.95–7.06 (m, 5H), 7.57 (d, *J* = 8.8 Hz, 1H), 7.77 (dd, *J* = 5.4 Hz, 7.9 Hz, 1H), 8.08 (s, 1H), 8.43 (d, *J* = 8.3 Hz, 1H), 9.36 (d, *J* = 4.4 Hz, 1H), 10.11 (s, 1H). ^13^C NMR (100.6 MHz, CDCl_3_) δ 116.37 (*J*_C−F_ = 23.1 Hz), 119.15, 119.40 (*J*_C−F_ = 34.2 Hz), 120.86, 122.32, 125.90, 126.23, 128.09, 133.80, 149.54, 157.27, 152.92, 153.17, 158.80 (*J*_C−F_ = 241.4 Hz), 160.15. HRMS (ESI) *m/z* calculated for C_17_H_11_ClFN_3_NaO_2_ [M+Na]^+^: 366.0416, found: 366.0420.

*N*-(3-chloro-4-(4-fluorophenoxy) phenyl) pyrazine-2-carboxamide (**4c**)

White solid, mp: 155.5–156.0 °C, ^1^H NMR (400 MHz, CDCl_3_) δ 6.92–7.05 (m, 5H), 7.59 (dd, *J* = 2.4 Hz, 8.8 Hz, 1H), 7.99 (d, *J* = 2.4 Hz, 1H), 8.60 (s, 1H), 8.84 (d, *J* = 2.2 Hz, 1H), 9.51 (s, 1H), 9.67 (s, 1H). ^13^C NMR (100.6 MHz, CDCl_3_) δ 116.35 (*J*_C−F_ = 23.1 Hz), 119.10, 119.33 (*J*_C−F_ = 27.2 Hz), 120.86, 122.18, 126.20, 133.85, 142.40, 143.98, 144.72, 147.81, 149.45, 152.92, 158.79 (*J*_C−F_ = 242.4 Hz), 160.67. HRMS (ESI) *m/z* calculated for C_18_H_13_ClFN_2_O_2_ [M+H]^+^: 343.0644, found: 343.0638.

*N*-(3-chloro-4-(4-fluorophenoxy) phenyl) nicotinamide (**4d**)

White solid, mp: 112.0–113.2 °C, ^1^H NMR (400 MHz, CDCl_3_) δ 6.89–6.93 (m, 3H), 7.02 (t, *J* = 9.1 Hz, 2H), 7.38–7.41 (m, 1H), 7.46 (dd, *J* = 2.5 Hz, 8.8 Hz, 1H), 7.83 (d, *J* = 2.4 Hz, 1H), 8.18 (dt, *J* = 1.8 Hz, 8.0 Hz, 1H), 8.71 (dd, *J* = 1.5 Hz, 4.8 Hz, 1H), 9.05 (s, 1H). ^13^C NMR (100.6 MHz, CDCl_3_) δ 116.38 (*J*_C−F_ = 23.1 Hz), 118.85, 119.26 (*J*_C−F_ = 8.0 Hz), 120.49 (*J*_C−F_ = 12.1 Hz), 123.15, 123.81, 125.91, 130.43, 130.04, 135.57, 147.91, 149.70, 152.52, 152.77, 158.82 (*J*_C−F_ = 241.4 Hz), 164.20. HRMS (ESI) *m/z* calculated for C_17_H_12_ClFN_3_O_2_ [M+H]^+^: 344.0597, found: 343.0600.

*N*-(3-chloro-4-(4-fluorophenoxy) phenyl) isonicotinamide (**4e**)

White solid, mp: 57.0–58.5 °C, ^1^H NMR (400 MHz, CDCl_3_) δ 6.90–6.93 (m, 3H), 7.00–7.04 (m, 2H), 7.46 (dd, *J* = 2.5 Hz, 8.8 Hz, 1H), 7.68–7.70 (m, 2H), 7.83 (d, *J* = 2.5 Hz, 1H), 8.70–8.71 (m, 3H). ^13^C NMR (100.6 MHz, CDCl_3_) δ 116.41 (*J*_C−F_ = 24.1 Hz), 118.82, 119.35 (*J*_C−F_ = 9.1 Hz), 120.43 (*J*_C−F_ = 10.1 Hz), 123.12, 125.91, 133.74, 141.74, 149.92, 150.58, 152.68, 158.87 (*J*_C−F_ = 241.4 Hz), 164.10. HRMS (ESI) *m/z* calculated for C_18_H_13_ClFN_2_O_2_ [M+H]^+^: 343.0644, found: 343.0640.

*N*-(3-chloro-4-(4-fluorophenoxy) phenyl) pyridazine-4-carboxamide (**4f**)

White solid, mp: 164.2–165.2 °C, ^1^H NMR (400 MHz, DMSO-*d_6_*) δ 7.02 (s, 2H), 7.16–7.23 (m, 3H), 7.69–7.79 (m, 1H), 8.11 (d, *J* = 6.1 Hz, 2H), 9.52 (d, *J* = 4.6 Hz, 1H), 9.65 (s, 1H), 10.89 (s, 1H). ^13^C NMR (100.6 MHz, DMSO-*d_6_*) δ 117.07 (*J*_C−F_ = 23.1 Hz), 119.40 (*J*_C−F_ = 8.0 Hz), 121.19, 121.88, 122.61 (*J*_C−F_ = 11.1 Hz), 124.90, 125.01, 132.39, 135.97, 148.45, 149.34, 152.62, 153.35, 158.46 (*J*_C−F_ = 225.3 Hz), 162.98.

*N*-(3-chloro-4-(3-fluorophenoxy) phenyl) picolinamide (**4g**)

White solid, mp: 79.0–80.0 °C, ^1^H NMR (400 MHz, CDCl_3_) δ 6.65 (dt, *J* = 2.4 Hz, 10.2 Hz, 1H), 6.72 (dd, *J* = 2.2 Hz, 8.3 Hz, 1H), 6.75–6.80 (m, 1H), 7.09 (d, *J* = 8.8 Hz, 1H), 7.23–7.28 (m, 1H), 7.48–7.51 (m, 1H), 7.64 (dd, *J* = 2.6 Hz, 8.8 Hz, 1H), 7.92 (td, *J* = 1.7 Hz, 7.7 Hz, 1H), 8.05 (d, *J* = 2.6 Hz, 1H), 8.29 (d, *J* = 7.8 Hz, 1H), 8.61 (dt, *J* = 0.64 Hz, 4.12 Hz, 1H), 10.08 (s, 1H). ^13^C NMR (100.6 MHz, CDCl_3_) δ 104.73 (*J*_C−F_ = 25.2 Hz), 109.76 (*J*_C−F_ = 21.1 Hz), 112.49 (*J*_C−F_ = 3.0 Hz), 119.36, 121.91, 122.44, 122.50, 126.77, 127.05, 130.53 (*J*_C−F_ = 10.1 Hz), 135.34, 137.82, 147.49, 148.07, 149.30, 158.83 (*J*_C−F_ = 10.1 Hz), 162.07, 163.55 (*J*_C−F_ = 246.5 Hz). HRMS (ESI) *m/z* calculated for C_17_H_12_ClFN_3_O_2_ [M+H]^+^: 344.0597, found: 344.0601.

*N*-(3-chloro-4-(3-fluorophenoxy) phenyl) pyridazine-3-carboxamide (**4h**)

White solid, mp: 130.3–131.2 °C, ^1^H NMR (400 MHz, CDCl_3_) δ 6.65–6.82 (m, 3H), 7.12 (d, *J* = 8.8 Hz, 1H), 7.27 (dd, *J* = 8.0 Hz, 15.0 Hz, 1H), 7.62 (dd, *J* = 1.8 Hz, 8.6 Hz, 1H), 7.75–7.79 (m, 1H), 8.10 (d, *J* = 1.8 Hz, 1H), 8.44 (d, *J* = 8.3 Hz, 1H), 9.37 (d, *J* = 4.3 Hz, 1H), 10.16 (s, 1H). ^13^C NMR (100.6 MHz, CDCl_3_) δ 104.90 (*J*_C−F_ = 25.2 Hz), 109.93 (*J*_C−F_ = 21.1 Hz), 112.64 (*J*_C−F_ = 3.0 Hz), 119.62, 120.33, 121.61, 122.33 (*J*_C−F_ = 9.1 Hz), 125.91, 127.13, 128.09, 130.55 (*J*_C−F_ = 10.1 Hz), 134.60, 148.13, 152.22, 153.19, 160.19, 160.5 (*J*_C−F_ = 366.2 Hz).

*N*-(3-chloro-4-(3-fluorophenoxy) phenyl) pyrazine-2-carboxamide (**4i**)

White solid, mp: 156.6–157.2 °C, ^1^H NMR (400 MHz, DMSO-*d_6_*) δ 6.75 (d, *J* = 8.4 Hz, 1H), 6.83 (d, *J* = 10.3 Hz, 1H), 6.96 (t, *J* = 8.2 Hz, 1H), 7.30 (d, *J* = 8.9 Hz, 1H), 7.41 (dd, *J* = 8.2 Hz, 15.4 Hz, 1H), 7.95–7.97 (m, 1H), 8.28 (d, *J* = 2.0 Hz, 1H), 8.85 (s, 1H), 8.97 (d, *J* = 2.1 Hz, 1H), 9.33 (s, 1H), 11.05 (s, 1H). ^13^C NMR (100.6 MHz, DMSO-*d_6_*) δ 104.75 (*J*_C−F_ = 25.2 Hz), 110.26 (*J*_C−F_ = 21.1 Hz), 112.87, 120.26, 121.45, 122.87 (*J*_C−F_ = 31.2 Hz), 125.56, 131.81 (*J*_C−F_ = 10.1 Hz), 136.70, 143.74, 144.63, 145.21, 146.89, 148.37, 158.88 (*J*_C−F_ = 11.1 Hz), 162.45, 163.38 (*J*_C−F_ = 244.5 Hz). HRMS (ESI) *m/z* calculated for C_17_H_11_ClFN_3_NaO_2_ [M+Na]^+^: 366.0416, found: 366.0415.

*N*-(3-chloro-4-(3-fluorophenoxy) phenyl) nicotinamide (**4j**)

White solid, mp: 117.3–118.2 °C, ^1^H NMR (400 MHz, MeOD) δ 6.63 (dt, *J* = 2.3 Hz, 10.4 Hz, 1H), 6.69 (dd, *J* = 2.3 Hz, 8.4 Hz, 1H), 6.81 (td, *J* = 1.9 Hz, 8.4 Hz, 1H), 7.12 (d, *J* = 8.8 Hz, 1H), 7.27–7.32 (m, 1H), 7.55–7.59 (m, 1H), 7.65 (dd, *J* = 2.5 Hz, 8.8 Hz, 1H), 8.02 (d, *J* = 2.5 Hz, 1H), 8.34 (dt, *J* = 1.8 Hz, 8.1 Hz, 1H), 8.72 (dd, *J* = 1.5 Hz, 5.0 Hz, 1H), 9.09 (d, *J* = 1.5 Hz, 1H). HRMS (ESI) *m/z* calculated for C_18_H_13_ClFN_2_O_2_ [M+H]^+^: 343.0644, found: 343.0648.

*N*-(3-chloro-4-(3-fluorophenoxy) phenyl) isonicotinamide (**4k**)

White solid, mp: 49.8–51.0 °C, ^1^H NMR (400 MHz, CDCl_3_) δ 6.63 (d, *J* = 10.1 Hz, 1H), 6.70 (d, *J* = 8.2 Hz, 1H), 6.75–6.81 (m, 1H), 7.04 (t, *J* = 8.7 Hz, 1H), 7.26 (dd, *J* = 8.2 Hz, 14.9 Hz, 1H), 7.52–7.62 (m, 1H), 7.70 (d, *J* = 4.8 Hz, 2H), 7.88 (d, *J* = 1.8 Hz, 1H), 8.71 (d, *J* = 5.0 Hz, 2H), 8.81 (s, 1H). ^13^C NMR (100.6 MHz, CDCl_3_) δ 104.95 (*J*_C−F_ = 24.1 Hz), 110.07 (*J*_C−F_ = 21.1 Hz), 112.73, 120.24 (*J*_C−F_ = 25.2 Hz), 121.11, 122.10, 122.49, 123.02, 126.88, 130.61 (*J*_C−F_ = 10.1 Hz), 134.71, 148.43, 150.57, 158.43 (*J*_C−F_ = 10.1 Hz), 163.21 (*J*_C−F_ = 187.1 Hz), 164.74. HRMS (ESI) *m/z* calculated for C_18_H_13_ClFN_2_O_2_ [M+H]^+^: 343.0644, found: 343.0639.

*N*-(3-chloro-4-(3-fluorophenoxy) phenyl) pyridazine-4-carboxamide (**4l**)

Yellow solid, mp: 141.3–142.7 °C, ^1^H NMR (400 MHz, CDCl_3_) δ 6.64 (d, *J* = 10.1 Hz, 1H), 6.72 (d, *J* = 8.4 Hz, 1H), 6.80 (t, *J* = 8.1 Hz, 1H), 7.06 (d, *J* = 8.7 Hz, 1H), 7.27 (dd, *J* = 8.0 Hz, 15.0 Hz, 1H), 7.58–7.68 (m, 1H), 7.95–8.02 (m, 2H), 9.30 (d, *J* = 5.0 Hz, 1H), 9.59 (s, 1H), 9.82 (s, 1H). ^13^C NMR (100.6 MHz, CDCl_3_) δ 105.04 (*J*_C−F_ = 25.2 Hz), 110.15 (*J*_C−F_ = 21.1 Hz), 112.84 (*J*_C−F_ = 3.0 Hz), 120.07, 120.56, 122.05, 123.22, 125.23, 126.87, 130.63 (*J*_C−F_ = 10.1 Hz), 133.09, 134.57, 148.67, 151.96, 158.29, 162.13, 163.52 (*J*_C−F_ = 247.5 Hz). HRMS (ESI) *m/z* calculated for C_17_H_12_ClFN_3_O_2_ [M+H]^+^: 344.0597, found: 344.0596.

*N*-(3-chloro-4-(3-chlorophenoxy) phenyl) picolinamide (**4m**)

Brown solid, mp: 92.7–93.5 °C, ^1^H NMR (400 MHz, CDCl_3_) δ 6.82 (dd, *J* = 1.9 Hz, 8.3 Hz, 1H), 6.92 (t, *J* = 2.1 Hz, 1H), 7.03–7.08 (m, 2H), 7.22 (t, *J* = 8.1 Hz, 1H), 7.47–7.50 (m, 1H), 7.63 (dd, *J* = 2.5 Hz, 8.8 Hz, 1H), 7.90 (td, *J* = 1.6 Hz, 7.7 Hz, 1H), 8.05 (d, *J* = 2.5 Hz, 1H), 8.28 (d, *J* = 7.8 Hz, 1H), 8.59 (d, *J* = 4.5 Hz, 1H), 10.07. ^13^C NMR (100.6 MHz, CDCl_3_) δ 115.18, 119.38, 121.91, 122.35, 122.47, 123.09, 126.76, 126.99, 130.52, 135.11, 135.35, 137.80, 147.43, 148.06, 149.27, 158.27, 162.06. HRMS (ESI) *m/z* calculated for C_18_H_13_Cl_2_N_2_O_2_ [M+H]^+^: 359.0349, found: 359.0346.

*N*-(3-chloro-4-(3-chlorophenoxy) phenyl) pyridazine-3-carboxamide (**4n**)

Light brown solid, mp: 138.0–139.6 °C, ^1^H NMR (400 MHz, DMSO-*d_6_*) δ 6.90 (dd, *J* = 1.9 Hz, 8.2 Hz, 1H), 7.01 (t, *J* = 2.0 Hz, 1H), 7.18 (d, *J* = 8.0 Hz, 1H), 7.31 (d, *J* = 8.9 Hz, 1H), 7.40 (t, *J* = 8.1 Hz, 1H), 7.99–8.02 (m, 2H), 8.32–8.36 (m, 2H), 9.50 (dd, *J* = 1.4 Hz, 4.9 Hz, 1H), 11.40 (s, 1H). ^13^C NMR (100.6 MHz, DMSO-*d_6_*) δ 115.63, 116.98, 121.63, 122.86, 123.12, 123.46, 125.59, 126.67, 129.19, 131.96, 134.50, 136.81, 146.78, 153.40, 154.13, 158.49, 162.23. HRMS (ESI) *m/z* calculated for C_17_H_11_Cl_2_N_3_NaO_2_ [M+Na]^+^: 382.0121, found: 382.0123.

*N*-(3-chloro-4-(3-chlorophenoxy) phenyl) pyrazine-2-carboxamide (**4o**)

Light brown solid, mp: 153.2–154.6 °C, ^1^H NMR (400 MHz, DMSO-*d_6_*) δ 6.90 (d, *J* = 8.0 Hz, 1H), 7.00 (s, 1H), 7.19 (d, *J* = 8.1 Hz, 1H), 7.30 (d, *J* = 8.8 Hz, 1H), 7.40 (t, *J* = 8.0 Hz, 1H), 7.96 (dd, *J* = 2.0 Hz, 8.8 Hz, 1H), 8.28 (d, *J* = 1.8 Hz, 1H), 8.84 (s, 1H), 8.96 (s, 1H), 9.32 (s, 1H), 11.05 (s, 1H). ^13^C NMR (100.6 MHz, DMSO-*d_6_*) δ 115.61, 116.98, 121.47, 122.72, 123.08, 123.45, 125.59, 131.94, 134.50, 136.76, 143.73, 144.63, 145.17, 146.75, 148.37, 158.47, 162.43. HRMS (ESI) *m/z* calculated for C_17_H_12_Cl_2_N_3_O_2_ [M+H]^+^: 360.0301, found: 360.0304.

*N*-(3-chloro-4-(3-chlorophenoxy) phenyl) nicotinamide (**4p**)

White solid, mp: 141.0–141.5 °C, ^1^H NMR (400 MHz, DMSO-*d_6_*) δ 6.89 (dd, *J* = 1.6 Hz, 8.4 Hz, 1H), 6.99 (s, 1H), 7.18 (d, *J* = 9.0 Hz, 1H), 7.30 (d, *J* = 8.8 Hz, 1H), 7.40 (t, *J* = 8.2 Hz, 1H), 7.58–7.61 (m, 1H), 7.77 (dd, *J* = 2.4 Hz, 8.9 Hz, 1H), 8.14 (d, *J* = 2.4 Hz, 1H), 8.30 (d, *J* = 8.1 Hz, 1H), 8.78–8.79 (m, 1H), 9.12 (d, *J* = 1.6 Hz, 1H), 10.66 (s, 1H). ^13^C NMR (100.6 MHz, DMSO-*d_6_*) δ 115.61, 116.93, 121.10, 122.34, 123.23, 123.45, 124.04, 125.65, 130.68, 131.96, 134.51, 135.97, 137.39, 146.53, 149.16, 152.83, 158.53, 164.73. HRMS (ESI) *m/z* calculated for C_18_H_13_Cl_2_N_2_O_2_ [M+H]^+^: 359.0349, found: 359.0351.

*N*-(3-chloro-4-(3-chlorophenoxy) phenyl) isonicotinamide (**4q**)

White solid, mp: 124.3–126.0 °C, ^1^H NMR (400 MHz, DMSO-*d_6_*) δ 6.91–7.00 (m, 2H), 7.19 (s, 1H), 7.28–7.40 (m, 2H), 7.78–7.88 (m, 3H), 8.15 (s, 1H), 8.82 (s, 2H), 10.72 (s, 1H).

*N*-(3-chloro-4-(3-chlorophenoxy) phenyl) pyridazine-4-carboxamide (**4r**)

Yellow solid, mp: 133.2–134.0 °C, ^1^H NMR (400 MHz, DMSO-*d_6_*) δ 6.91 (dd, *J* = 2.2 Hz, 8.3 Hz, 1H), 7.01 (s, 1H), 7.20 (d, *J* = 8.0 Hz, 1H), 7.32 (d, *J* = 8.8 Hz, 1H), 7.42 (t, *J* = 8.2 Hz, 1H), 7.76 (dd, *J* = 2.4 Hz, 8.9 Hz, 1H), 8.13 (d, *J* = 2.3 Hz, 2H), 9.53 (d, *J* = 5.2 Hz, 1H), 9.67 (s, 1H), 10.92 (s, 1H). ^13^C NMR (100.6 MHz, DMSO-*d_6_*) δ 115.74, 117.09, 121.22, 122.49, 123.18, 123.57, 125.01, 125.65, 131.97, 132.34, 134.53, 136.74, 147.03, 149.32, 152.62, 158.38, 163.04. HRMS (ESI) *m/z* calculated for C_17_H_12_Cl_2_N_3_O_2_ [M+H]^+^: 360.0301, found: 360.0304.

### 3.2. Biological Evaluation

#### 3.2.1. In Vitro Kinase Screening

Kinase assays were performed at the Reaction Biology Corporation (Malvern, PA) using the “HotSpot” assay platform. All the target compounds were evaluated according to the Reaction Biology Corp implemented protocol as reported on their website (http://www.reactionbiology.com/ 25 January 2019).

#### 3.2.2. NCI Cell Line Screening

The target compounds were evaluated for their anti-proliferative activity at the National Cancer Institute (NCI), Bethesda, Maryland, USA, applying the standard protocol of the NCI (http://www.dtp.nci.nih.gov/).

#### 3.2.3. Docking Methodology

The X-ray crystal structures of DAPK1 kinase in complex with Genistein (PDB ID: 5AUZ) were downloaded from the protein data bank (www.rcsb.org) in PDB format. The 2D structure of compound **4j** was drawn using ChemDraw software. Molecular Operating Environment (MOE, 2014.0901) software was used for the molecular docking operation docking protocol. DAPK1 kinase was prepared for the molecular docking procedure by applying 3D protonation of both enzyme amino acids and the native ligand (Genistein). In addition, water from crystallization was removed from the kinase domain. The active site was isolated. The grid space was determined from the coordinates of the reference ligand (−22.780412, 2.367623, −10.682060) and expanded to cover all amino acid residues in the DAPK1 active site cavity (r = 8.383472). The docking protocol was validated by re-docking of Genistein with RMSD = 0.8894 Å and retained the same binding pose in the DAPK1 active site.

## 4. Conclusions

In the current study, a series of 3-chloro-4-(phenoxy)phenyl carboxamide derivatives featuring different substituents on the terminal phenoxy group, along with diverse nitrogen containing heterocycles on the carboxamide linker, has been designed and synthesized as DAPK1 inhibitors with potential anti-proliferative activity. A preliminary kinase inhibition evaluation was conducted on the first synthesized compound **4a** in an attempt to explore its DAPK1 activity and selectivity. Among the 45 panel kinases, compound **4a** exhibited higher activity and selectivity against DAPK1 kinase. Hence, DAPK1 inhibitory activity of the synthesized derivatives was detected; pyridinyl moiety owing derivatives **4h**, **4j**, **4f**, and **4q** displayed the highest activity and were subjected to 10-dose evaluation. The IC_50_ values of the mentioned compounds were determined and compound **4q** emerged to be the most potent DAPK1 inhibitor (IC_50_ =1.09 µM). Additionally, all the target compounds were evaluated for their cytotoxic activity against a panel of 60 NCI cancer cell lines at 10 µM concentration. Out of the tested series, compounds **4d**, **4e**, **4o**, and **4p** revealed the highest anti-proliferative activities against most of the employed cell lines (67.02%, 67.87%, 70.83%, and 69.27%. Moreover, two leukemia cell lines (HL-60(TB) and K-562) exhibited the highest sensitivity towards the tested candidates. 

## Figures and Tables

**Table 1:** Key Structure of the final target compounds **4a**–**r**.

Cpd	R	Ar	Yield%	Cpd	R	Ar	Yield%
**4a**	4-F	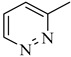	73	**4j**	4-F	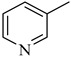	70
**4b**	3-F	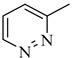	50	**4k**	4-F	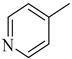	56
**4c**	3-F	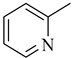	63	**4l**	4-F	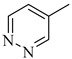	46
**4d**	3-F	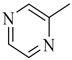	59	**4m**	3-Cl	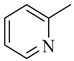	62
**4e**	3-F	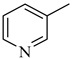	23	**4n**	3-Cl	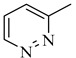	72
**4f**	3-F	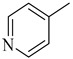	29	**4o**	3-Cl	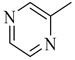	65
**4g**	3-F	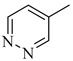	62	**4p**	3-Cl	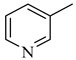	49
**4h**	4-F	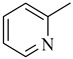	82	**4q**	3-Cl	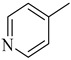	12
**4i**	4-F	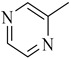	86	**4r**	3-Cl	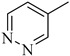	53

**Table 2:** % enzyme inhibition (relative to DMSO controls) of compound **4a** in a single-dose duplicate mode assay at a concentration of 10 µM against 45 kinases.

Kinase	DAPK1% Inhibition at 10 μM Dose ^a^	Kinase	DAPK1% Inhibition at 10 μM Dose ^a^
ABL1	12.29 ± 0.11	JAK1	8.11 ± 1.85
ACK1	0.53 ± 3.32	JNK1	−9.39 ± 0.22
AKT1	−6.56 ± 1.56	KDR/VEGFR2	−4.28 ± 0.11
AMPK(A1/B1/G1)	2.10 ± 2.96	LCK	−15.69 ± 0.45
Aurora A	−5.10 ± 0.78	LRRK2	23.80 ± 1.19
BRAF	−4.67 ± 0.03	MARK1	−3.12 ± 0.15
BRAF (V599E)	−9.72 ± 0.04	MEK1	−16.18 ± 0.42
BTK	1.97 ± 0.21	OSR1/OXSR1	−6.76 ± 1.93
CAMK1a	−49.53 ± 0.28	P38a/MAPK14	−22.83 ± 0.63
CDK1/cyclin A	−8.34 ± 0.93	PAK1	1.12 ± 2.73
CHK1	−7.21 ± 0.77	PDGFRa	19.18 ± 0.68
c-Kit	15.53 ± 3.14	RAF1	4.51 ± 1.32
CLK1	−0.14 ± 0.13	RET	−12.78 ± 0.99
c-MET	7.03 ± 0.34	ROCK1	−6.35 ± 1.35
c-Src	10.63 ± 1.61	ROCK2	2.96 ± 1.06
DAPK1	44.19 ± 1.95	ROS/ROS1	−7.04 ± 1.71
DDR1	5.76 ± 1.69	STK39/STLK3	−2.47 ± 1.50
ERBB2/HER2	−7.42 ± 1.74	SYK	−13.26 ± 0.10
FGFR1	−14.24 ± 0.24	TAK1	−4.08 ± 1.16
FLT3	0.13 ± 1.08	TIE2/TEK	−13.18 ± 2.15
FMS	3.36 ± 0.31	TLK1	−2.23 ± 3.33
IGF1R	−12.67 ± 0.72	TRKA	−8.71 ± 0.17
IKKb/IKBKB	−15.92 ± 0.74		

^a^ Inhibition percentage values expressed as a mean of duplicate measurements ± S.E.M.

**Table 3:** DAPK1 kinase % inhibition activity for the final target compounds **4a**–**r** at 10 µM.

Cpd	% Inhibition against DAPK1 ^a^	Cpd	% Inhibition against DAPK1 ^a^
**4a**	44.19	**4j**	79.06
**4b**	58.95	**4k**	80.61
**4c**	58.83	**4l**	61.83
**4d**	50.91	**4m**	61.73
**4e**	59.98	**4n**	61.54
**4f**	64.06	**4o**	37.99
**4g**	50.18	**4p**	61.48
**4h**	81.00	**4q**	72.10
**4i**	63.69	**4r**	59.98

^a^ % enzyme inhibition (relative to DMSO controls) for all synthesized analogs in a single-dose duplicate mode assay at a concentration of 10 µM with DAPK1 kinase.

**Table 4:** IC_50_ values of compounds **4h**, **4j**, **4k**, and **4q** against DAPK1 kinase.

Cpd	DAPK1 ^a^, IC_50_ (μM)
**4h**	6.81
**4j**	1.70
**4k**	7.26
**4q**	1.09

^a^ Mean values of duplicate mode 10-dose IC_50_ assays with DAPK1 kinase, with 3-fold serial dilution starting at 10 μM.

**Table 5:** Anti-proliferative activities (% growth inhibition) of the target compounds **4a**–**r** against leukemia cell lines.

Cpd.	CCRF-CEM	HL-60(TB)	K-562	MOLT-4	RPMI-8226	SR
**4a**	24.87	43.74	44.47	40.16	37.38	2.35
**4b**	24.49	26.45	38.23	19.72	42.72	−6.10
**4c**	6.71	11.44	10.98	6.93	21.08	−4.05
**4d**	81.59	96.88	86.57	58.79	82.05	79.23
**4e**	76.04	87.94	83.79	59.83	84.21	76.25
**4f**	41.83	32.93	67.22	27.56	54.45	36.39
**4g**	40.77	30.04	44.68	24.07	55.39	8.58
**4h**	−5.45	−5.22	4.80	−24.34	−1.85	−26.54
**4i**	35.87	78.57	78.88	54.90	19.59	50.71
**4j**	−0.75	16.79	49.09	-5.20	12.69	24.85
**4k**	21.90	16.39	53.90	5.43	34.21	10.68
**4l**	21.39	15.06	19.57	12.50	33.55	−2.96
**4m**	−0.92	12.35	−7.67	−12.52	0.39	−26.02
**4n**	16.03	10.46	23.01	19.89	28.89	13.01
**4o**	83.35	98.50	86.78	73.85	81.94	73.68
**4p**	82.54	93.94	88.94	62.59	88.07	80.15
**4q**	43.23	40.60	71.93	32.16	59.20	18.60
**4r**	34.26	18.01	41.54	14.16	46.55	−11.83

**Table 6:** Anti-proliferative activities (% growth inhibition) of the target compounds **4a**–**r** against non-small cell lung cancer cell lines.

Cpd	A549/ ATCC	EKVX	HOP-62	HOP-92	NCI-H226	NCI-H23	NCI-H322M	NCI-H460	NCI-H522
**4a**	34.51	25.04	15.76	33.54	45.00	37.68	12.42	23.21	38.20
**4b**	32.62	25.29	0.90	17.89	30.57	23.57	6.18	3.78	27.10
**4c**	18.95	10.63	−7.22	15.05	1.09	6.75	2.94	0.03	17.87
**4d**	60.88	48.75	57.56	48.48	38.23	55.06	53.70	77.90	77.71
**4e**	64.48	50.41	65.97	36.59	43.08	55.14	49.18	75.35	63.79
**4f**	44.90	46.82	31.88	12.60	37.44	49.29	10.81	22.18	24.20
**4g**	36.08	35.47	10.18	NT	30.80	33.47	−0.14	11.06	22.76
**4h**	1.70	13.06	−8.34	7.68	5.54	14.36	1.66	0.15	20.06
**4i**	56.25	21.95	43.80	41.84	25.60	34.41	17.83	71.99	45.79
**4j**	14.04	18.08	−6.37	8.10	0.24	20.04	−0.70	3.01	27.70
**4k**	12.58	60.37	29.52	20.18	17.62	51.60	1.00	18.70	37.11
**4l**	24.02	19.22	1.57	16.91	26.86	25.71	7.09	5.29	20.81
**4m**	8.08	−0.64	−6.77	3.98	−8.86	−5.18	−5.68	−0.87	5.22
**4n**	24.70	12.67	−0.67	15.62	21.16	15.91	6.13	0.21	26.35
**4o**	76.05	46.82	59.01	37.99	35.91	53.91	52.07	84.28	87.52
**4p**	63.21	49.14	62.02	38.66	45.68	52.09	52.92	77.80	72.66
**4q**	50.61	47.56	33.07	13.96	42.84	55.74	19.25	28.11	35.65
**4r**	32.61	28.53	6.68	5.58	29.92	32.81	1.73	7.58	27.65

**Table 7:** Anti-proliferative activities (% growth inhibition) of the target compounds **4a**–**r** against colon adenocarcinoma cell lines.

Cpd	COLO 205	HCC-2998	HCT-116	HCT-15	HT29	KM12	SW-620
**4a**	−11.74	10.88	3.23	22.43	23.27	20.85	5.86
**4b**	−0.78	3.23	44.58	48.54	13.03	17.64	−3.69
**4c**	−10.09	7.89	14.49	24.71	15.35	4.65	−2.86
**4d**	84.52	43.81	67.97	68.78	90.87	69.66	77.31
**4e**	77.29	42.26	66.25	70.11	87.31	70.73	74.22
**4f**	9.29	−6.85	52.29	19.16	3.92	29.08	15.56
**4g**	1.75	−5.80	21.38	20.12	0.43	25.11	1.33
**4h**	−24.87	6.42	8.49	3.76	−3.35	1.13	5.68
**4i**	30.70	23.68	41.31	52.26	76.34	72.49	63.04
**4j**	−20.33	8.79	11.85	15.67	11.48	23.60	7.30
**4k**	4.83	27.39	56.60	13.33	1.72	22.04	14.96
**4l**	−2.63	3.36	6.44	9.12	12.54	10.52	−5.05
**4m**	−12.61	−17.45	−3.79	−0.91	−2.08	−1.10	−0.09
**4n**	−5.90	4.76	33.28	43.26	10.11	14.71	−4.84
**4o**	87.71	49.22	79.08	76.69	97.86	79.27	77.28
**4p**	84.61	42.04	70.09	63.16	91.43	71.89	74.59
**4q**	17.91	−0.82	55.73	24.37	15.41	32.88	23.45
**4r**	−1.57	−10.77	15.72	17.28	3.02	21.25	−0.48

**Table 8:** Anti-proliferative activities (% growth inhibition) of the target compounds **4a**–**r** against CNS cancer cell lines.

Cpd	SF-268	SF-295	SF-539	SNB-19	SNB-75	U251
**4a**	19.64	26.63	14.50	33.85	12.24	37.09
**4b**	15.24	19.22	3.77	13.63	19.94	30.61
**4c**	3.67	6.83	−4.47	8.03	20.26	21.79
**4d**	44.50	63.96	103.46	59.62	99.15	74.34
**4e**	46.35	59.88	95.94	57.35	92.15	67.54
**4f**	15.83	59.58	17.49	32.89	10.07	27.52
**4g**	18.94	22.54	7.93	18.58	14.60	21.98
**4h**	−1.32	8.92	4.25	8.73	NT	12.52
**4i**	27.80	27.12	32.90	36.26	52.28	61.85
**4j**	5.00	14.00	5.58	6.79	NT	10.97
**4k**	16.97	58.92	18.58	26.78	NT	14.64
**4l**	18.14	14.27	4.69	11.91	28.81	15.77
**4m**	−1.72	5.99	−3.28	4.33	12.89	9.88
**4n**	16.12	17.78	4.69	10.47	22.28	23.31
**4o**	45.68	58.77	135.24	66.47	120.94	83.53
**4p**	46.58	58.61	108.37	58.71	123.19	76.10
**4q**	20.45	67.42	24.18	41.32	18.27	37.68
**4r**	17.40	27.73	4.39	18.63	12.21	19.46

**Table 9:** Anti-proliferative activities (% growth inhibition) of the target compounds **4a**–**r** against melanoma cell lines.

Cpd	LOX IMVI	MALME-3M	M14	MDA-MB-435	SK-MEL-2	SK-MEL-28	SK-MEL-5	UACC-257	UACC-62
**4a**	27.24	−4.65	−14.00	32.75	22.30	9.15	69.25	35.74	37.47
**4b**	25.22	7.97	7.10	19.00	8.32	1.43	32.67	7.10	31.60
**4c**	2.62	2.27	−1.30	8.65	5.50	−0.68	9.49	−9.00	16.27
**4d**	56.13	48.64	74.39	121.17	62.33	39.39	90.39	35.06	66.46
**4e**	47.19	52.84	84.06	98.36	63.46	45.96	123.95	43.92	68.80
**4f**	38.68	16.33	15.91	33.96	30.20	13.40	58.84	41.05	48.32
**4g**	18.38	−3.55	11.13	23.66	14.50	2.85	41.79	19.72	36.67
**4h**	3.90	−1.10	−5.94	5.26	−4.51	−14.37	2.21	−12.41	12.42
**4i**	48.55	29.89	7.62	102.80	46.40	23.13	50.48	20.25	49.67
**4j**	8.76	−2.94	2.72	58.51	8.69	−2.54	20.48	8.59	18.73
**4k**	29.89	8.49	6.80	28.75	40.51	1.54	67.41	23.72	40.20
**4l**	10.38	−2.67	5.36	10.47	2.21	−0.94	21.76	6.33	25.61
**4m**	-2.57	−9.79	−4.28	6.16	−7.38	0.87	−3.94	−4.90	6.68
**4n**	11.48	−3.78	7.23	22.44	−2.18	0.55	16.20	−3.76	25.33
**4o**	70.14	46.63	80.21	139.73	56.37	35.07	74.19	27.57	71.64
**4p**	48.29	49.87	78.15	144.28	55.91	36.16	85.47	35.81	62.37
**4q**	44.43	27.35	26.33	40.27	35.39	17.55	73.04	38.68	49.14
**4r**	14.41	2.90	15.11	21.71	14.60	5.10	29.20	16.23	33.19

**Table 10:** Anti-proliferative activities (% growth inhibition) of the target compounds **4a**–**r** against ovarian cancer cell lines.

Cpd	IGROV1	OVCAR-3	OVCAR-4	OVCAR-5	OVCAR-8	NCI/ADR-RES	SK-OV-3
**4a**	2.39	26.40	NT	15.95	28.75	30.57	11.16
**4b**	7.95	21.21	38.86	3.48	6.93	7.85	−3.63
**4c**	5.29	5.30	21.99	2.65	−2.78	−3.02	−7.73
**4d**	59.49	117.85	55.46	43.39	62.49	90.94	14.03
**4e**	58.24	93.03	53.83	27.40	65.37	86.14	69.98
**4f**	10.32	24.90	54.30	−3.67	33.96	30.30	18.97
**4g**	−0.70	18.15	26.00	−8.04	11.94	8.76	−1.53
**4h**	1.79	−2.65	11.49	−5.31	−5.64	11.70	−14.73
**4i**	37.38	56.36	NT	18.94	28.56	59.16	−0.47
**4j**	4.59	14.21	14.85	0.07	1.40	14.87	−10.36
**4k**	7.73	31.85	22.92	7.00	27.11	36.38	19.08
**4l**	3.35	12.54	24.55	−2.71	9.85	7.17	3.83
**4m**	−4.42	−2.87	1.25	6.70	−3.01	−14.69	−3.50
**4n**	8.57	14.44	31.35	8.86	8.33	15.03	−2.30
**4o**	72.96	107.23	61.05	39.64	72.05	85.16	16.73
**4p**	57.85	97.34	52.16	28.80	67.92	88.11	65.75
**4q**	14.33	27.51	56.18	9.72	40.42	33.17	19.67
**4r**	7.86	14.64	31.04	−5.97	10.63	4.76	0.32

**Table 11:** Anti-proliferative activities (% growth inhibition) of the target compounds **4a**–**r** against renal cancer cell lines.

Cpd	786-0	A498	ACHN	CAKI-1	RXF 393	SN12C	TK-10	UO-31
**4a**	−5.04	38.95	26.30	45.20	17.36	36.18	35.56	45.01
**4b**	23.45	21.77	14.60	24.59	20.89	15.74	25.76	33.84
**4c**	21.26	10.29	2.04	14.58	−4.88	7.62	22.97	17.68
**4d**	53.47	30.57	62.30	74.84	92.63	54.42	40.46	56.60
**4e**	59.13	95.84	64.82	67.45	86.46	63.63	40.07	60.28
**4f**	23.38	41.23	44.98	33.92	−6.80	18.18	22.50	31.83
**4g**	15.44	24.78	31.68	37.07	0.38	18.44	18.94	31.99
**4h**	5.85	22.23	−2.59	15.45	−0.44	−4.70	1.08	22.52
**4i**	18.95	32.33	5.89	50.35	27.74	30.22	14.22	34.97
**4j**	7.73	29.16	0.47	13.15	9.82	4.16	2.30	19.09
**4k**	16.26	56.91	25.12	30.56	8.16	16.37	15.85	28.68
**4l**	13.93	19.59	12.22	22.93	−11.93	14.96	18.03	40.38
**4m**	9.93	5.35	−1.09	7.21	−11.85	2.65	7.95	12.70
**4n**	22.57	12.65	2.11	19.24	9.70	16.79	24.95	35.06
**4o**	55.36	49.81	60.04	78.37	104.58	55.60	43.63	62.66
**4p**	60.79	103.10	58.34	69.44	100.14	56.56	40.23	55.92
**4q**	27.67	40.37	52.45	41.45	9.56	30.90	25.70	43.59
**4r**	14.13	27.83	26.38	33.64	−20.50	16.91	17.18	35.82

**Table 12:** Anti-proliferative activities (% growth inhibition) of the target compounds **4a**-**r** against prostate cancer cell lines.

Cpd	PC-3	DU-145
**4a**	35.59	23.29
**4b**	47.62	17.49
**4c**	33.62	8.58
**4d**	83.31	80.87
**4e**	75.41	78.17
**4f**	20.74	9.42
**4g**	28.39	16.44
**4h**	2.71	3.23
**4i**	23.96	20.47
**4j**	9.30	12.96
**4k**	24.42	22.70
**4l**	13.07	5.67
**4m**	9.03	−3.53
**4n**	42.83	13.68
**4o**	88.90	77.35
**4p**	85.15	83.88
**4q**	25.02	22.55
**4r**	19.59	15.68

**Table 13:** Anti-proliferative activities (% growth inhibition) of the target compounds **4a**–**r** against breast cancer cell lines.

Cpd	MCF7	MDA-MB-231/ATCC	HS 578T	BT-549	T-47D	MDA-MB-468
**4a**	34.71	11.88	4.36	−3.11	62.39	60.73
**4b**	12.80	6.93	−4.53	15.34	42.13	41.58
**4c**	11.41	−0.05	1.61	7.71	23.18	14.77
**4d**	82.54	46.50	72.17	40.15	61.71	99.92
**4e**	80.44	39.78	68.75	51.44	66.51	109.22
**4f**	44.11	14.71	7.30	40.61	46.75	76.00
**4g**	18.46	1.84	8.39	8.12	39.23	55.16
**4h**	13.86	0.09	−0.49	2.31	27.84	20.32
**4i**	70.32	41.29	37.62	−0.40	57.08	68.17
**4j**	23.32	6.34	8.51	7.52	29.40	42.77
**4k**	39.38	19.70	6.71	36.00	56.60	78.17
**4l**	9.28	9.19	−7.24	5.75	30.08	36.63
**4m**	3.69	−2.42	3.47	1.02	−4.39	−2.35
**4n**	8.35	4.49	−6.22	1.47	27.64	36.69
**4o**	79.69	68.29	81.27	42.55	59.27	99.57
**4p**	78.44	41.02	68.95	46.72	64.41	109.21
**4q**	56.26	20.80	10.84	40.15	51.56	74.71
**4r**	14.03	5.90	2.82	1.87	38.96	52.01

## Data Availability

Data is contained within the article and supplementary material.

## Supplementary Materials

The following are available online at: https://www.mdpi.com/article/10.3390/ph15091050/s1.
