# Supplementary material for: Identification of Novel Aryl Carboxamide Derivatives as Death-Associated Protein Kinase 1 (DAPK1) Inhibitors with Anti-Proliferative Activities: Design, Synthesis, In Vitro, and In Silico Biological Studies
Source: Pharmaceuticals (Basel). 2022 Aug 25;15(9):1050. doi: 10.3390/ph15091050 (PMC9504985; doi:10.3390/ph15091050)
Supplement: Supplementary file 1 [file pharmaceuticals-15-01050-s001.zip › pharmaceuticals-1783768-supplementary.pdf]

Supplementary Material

2D-NMR representative spectrum of compound **4a**

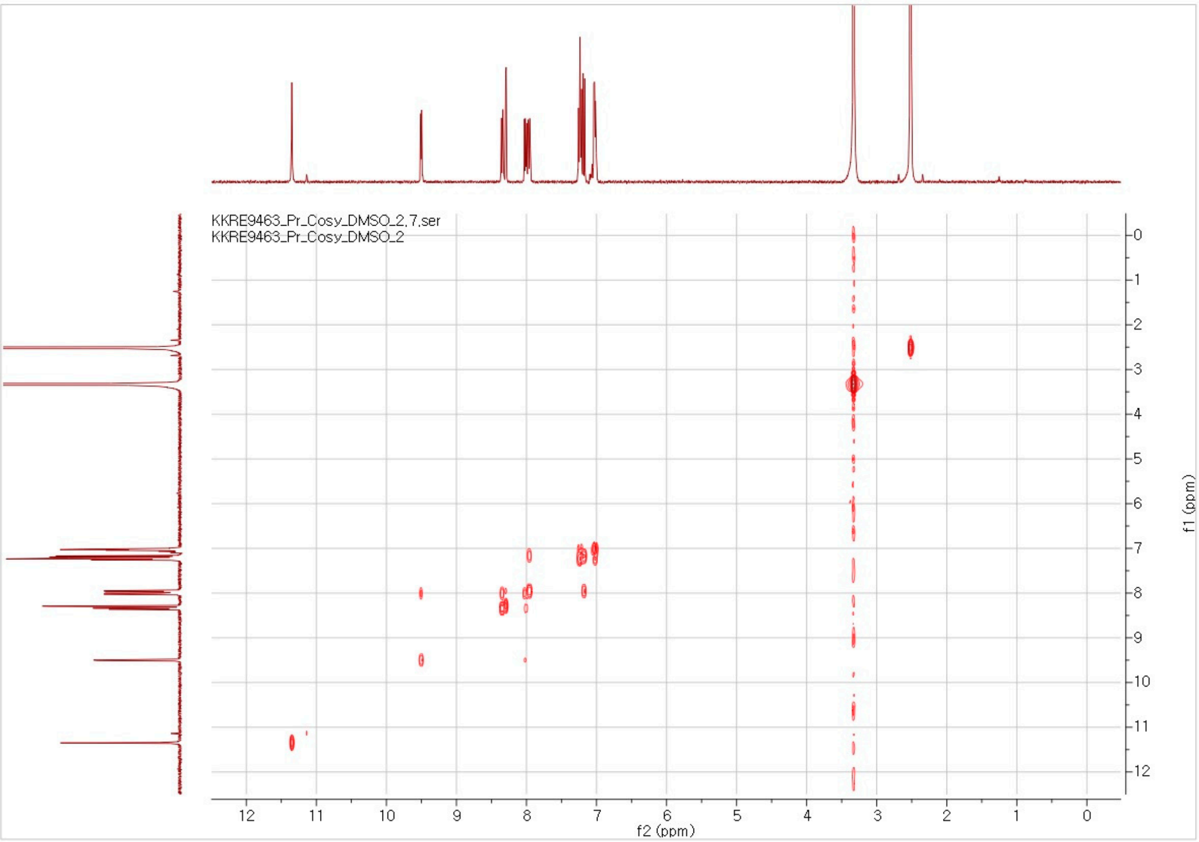

HRMS chat of compound **4a**

Acquisition Parameter

Source Type	ESI	Ion Polarity	Positive	Set Nebulizer	0.3 Bar
Focus	Active	Set Capillary	4500 V	Set Dry Heater	200 °C
Scan Begin	50 m/z	Set End Plate Offset	-500 V	Set Dry Gas	4.0 l/min
Scan End	600 m/z	Set Charging Voltage	2000 V	Set Divert Valve	Source
		Set Corona	0 nA	Set APCI Heater	0 °C

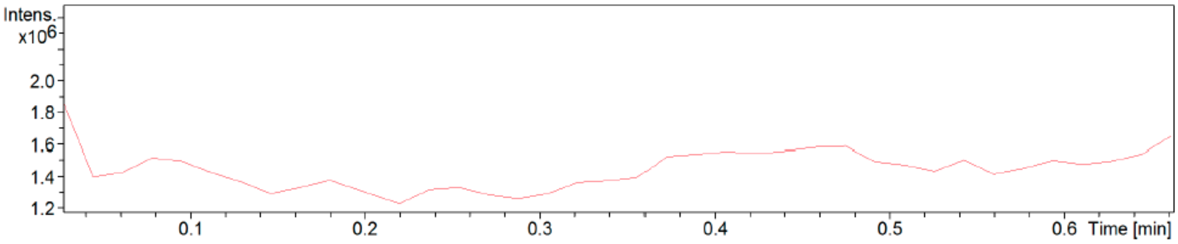

#	RT [min]	Area	Int. Type	I	S/N	Chromatogram	Max. m/z	FWHM [min]
n.a.	0.4	n.a.	Average spectrum	n.a.	n.a.	n.a.	366.0408	n.a.

+MS, 0.2-0.6min #10-36

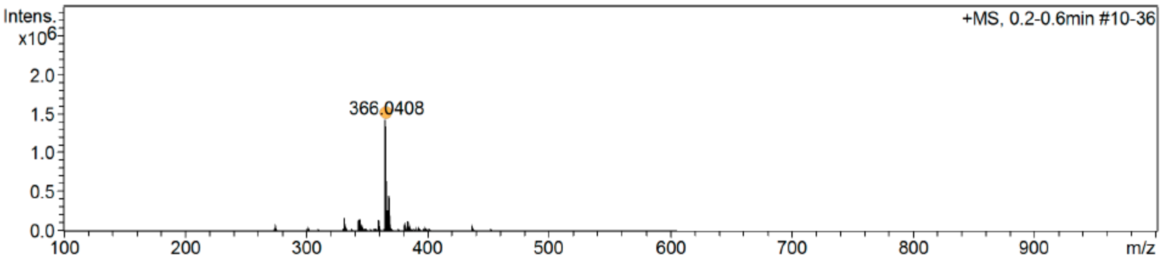

Meas. m/z	#	Ion Formula	m/z	err [ppm]	mSigma	# Sigma	Score	rdb	e⁻ Conf	N-Rule
366.0408	1	C17H11ClFN3NaO2	366.0416	-2.1	8.4	1	100.00	12.5	even	ok
	2	C15H10ClF4N3Na	366.0392	4.6	9.2	2	57.18	9.5	even	ok

HRMS chat of compound **4b**

Acquisition Parameter

Source Type	ESI	Ion Polarity	Positive	Set Nebulizer	0.3 Bar
Focus	Active	Set Capillary	4500 V	Set Dry Heater	200 °C
Scan Begin	50 m/z	Set End Plate Offset	-500 V	Set Dry Gas	4.0 l/min
Scan End	425 m/z	Set Charging Voltage	2000 V	Set Divert Valve	Source
		Set Corona	0 nA	Set APCI Heater	0 °C

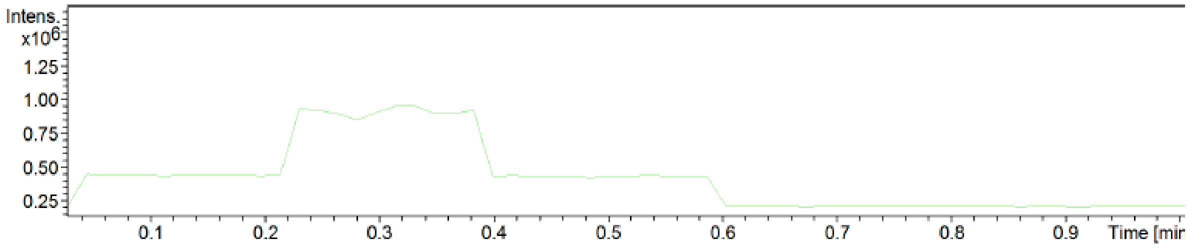

#	RT [min]	Area	Int. Type	I	S/N	Chromatogram	Max. m/z	FWHM [min]
n.a.	0.7	n.a.	Average spectrum	n.a.	n.a.	n.a.	366.0420	n.a.

+MS, 0.6-0.8min #34-47

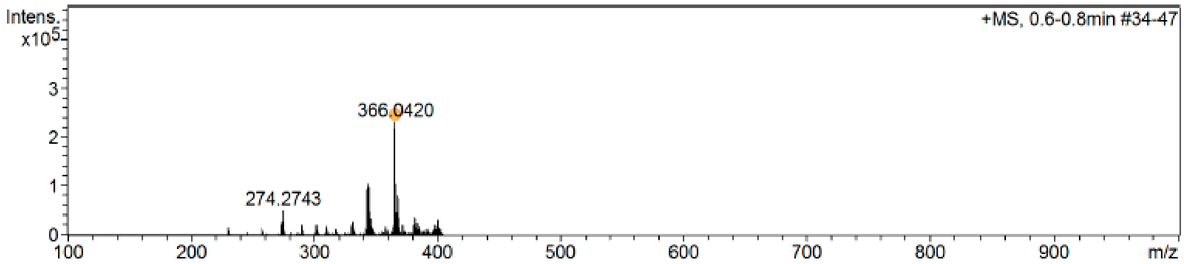

Meas. m/z	#	Ion Formula	m/z	err [ppm]	mSigma	# Sigma	Score	rdb	e⁻ Conf	N-Rule
366.0420	1	C15H6ClFN9	366.0413	-1.7	5.6	1	89.02	16.5	even	ok
	2	C17H9ClF4N3	366.0416	1.1	5.6	2	100.00	12.5	even	ok
	3	C19H10ClFN3O2	366.0440	-5.6	7.5	3	34.87	15.5	even	ok
	4	C16H11ClF2N3O3	366.0452	8.7	8.0	4	12.48	11.5	even	ok
	5	C14H10ClFN5O4	366.0400	-5.4	14.5	5	32.58	11.5	even	ok
	6	C14H10ClF5N3O	366.0427	2.1	19.3	6	64.23	8.5	even	ok
	7	C12H7ClF2N9O	366.0425	-1.4	19.3	7	72.51	12.5	even	ok
	8	C13H14ClFNO8	366.0386	-9.0	25.2	8	7.84	6.5	even	ok
	9	C13H15Cl2F5NO	366.0445	7.1	133.7	9	0.19	3.5	even	ok
	10	C14H13Cl2F3N3O	366.0382	10.2	134.4	10	0.06	7.5	even	ok
	11	C16H14Cl2F4N	366.0434	3.9	134.5	11	0.44	7.5	even	ok
	12	C14H11Cl2FN7	366.0432	-3.3	134.5	12	0.51	11.5	even	ok
	13	C13H15Cl2FN3O4	366.0418	0.4	136.9	13	0.76	6.5	even	ok
	14	C18H15Cl2FNO2	366.0458	10.6	139.1	14	0.04	10.5	even	ok
	15	C13H18Cl3F3NO	366.0401	-5.2	249.4	15	0.00	2.5	even	ok
	16	C13H16Cl3FN5	366.0450	-8.3	249.8	16	0.00	6.5	even	ok
	17	C12H20Cl3FNO4	366.0436	-4.6	251.7	17	0.00	1.5	even	ok
	18	C16H17Cl3F2N	366.0389	8.3	251.8	18	0.00	6.5	even	ok
	1	C17H11ClFN3NaO2	366.0416	-1.0	3.7	1	100.00	12.5	even	ok

HRMS chat of compound **4c**

Analysis Info

Analysis Name	D:\Data\Data\2022\KIST\NEJ\KSJ07\KSJ006.d	Acquisition Date	7/12/2022 5:19:41 PM
Method	Tune_pos_Standard_50_600_20210713.d.m	Operator	lee
Sample Name	6KKRE9468	Instrument	compact 8255754.10024
Comment			

Acquisition Parameter

Source Type	ESI	Ion Polarity	Positive	Set Nebulizer	0.3 Bar
Focus	Active	Set Capillary	4500 V	Set Dry Heater	200 °C
Scan Begin	50 m/z	Set End Plate Offset	-500 V	Set Dry Gas	4.0 l/min
Scan End	400 m/z	Set Charging Voltage	2000 V	Set Divert Valve	Source
		Set Corona	0 nA	Set APCI Heater	0 °C

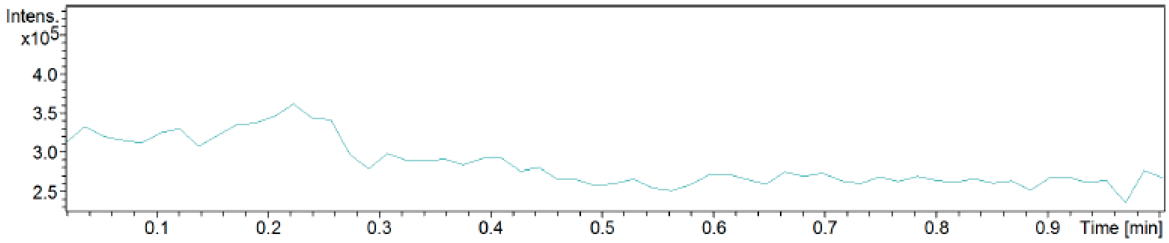

#	RT [min]	Area	Int. Type	I	S/N	Chromatogram	Max. m/z	FWHM [min]
n.a.	0.4	n.a.	Average spectrum	n.a.	n.a.	n.a.	343.0638	n.a.

+MS, 0.0-0.7min #2-43

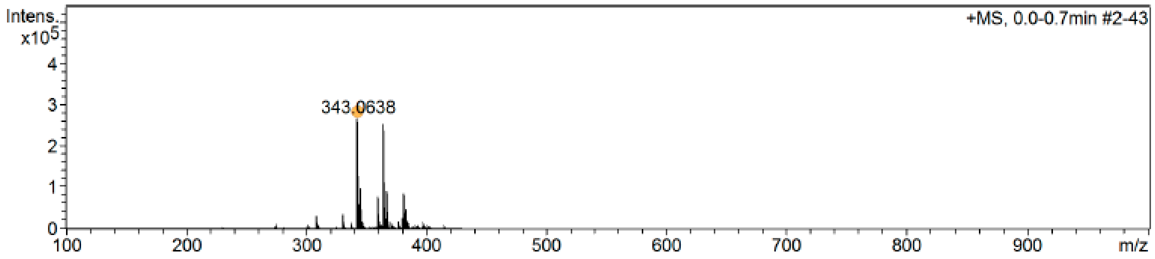

Meas. m/z	#	Ion Formula	m/z	err [ppm]	mSigma	# Sigma	Score	rdb	e⁻ Conf	N-Rule
343.0638	1	C18H13ClFN2O2	343.0644	-1.7	26.5	1	100.00	12.5	even	ok
	2	C14H9ClFN8	343.0617	6.1	33.6	2	32.41	13.5	even	ok
	3	C16H12ClF4N2	343.0620	-5.4	33.6	3	38.88	9.5	even	ok
	4	C15H14ClF2N2O3	343.0656	-5.1	34.9	4	40.82	8.5	even	ok
	5	C15H17Cl2F4	343.0638	-0.1	121.2	5	4.00	4.5	even	ok
	6	C14H19Cl2F2O3	343.0674	10.4	123.8	6	0.26	3.5	even	ok
	7	C17H18Cl2FO2	343.0662	7.1	124.2	7	0.77	7.5	even	ok
	1	C21H14ClFNa	343.0660	-6.4	25.3	1	86.56	13.5	even	ok
	2	C18H15ClF2NaO	343.0672	9.8	28.8	2	27.76	9.5	even	ok
	3	C16H14ClFN2NaO2	343.0620	-5.3	32.1	3	100.00	9.5	even	ok
	4	C15H19Cl2FNaO2	343.0638	-0.1	123.0	4	8.68	4.5	even	ok

HRMS chat of compound **4d**

Acquisition Parameter

Source Type	ESI	Ion Polarity	Positive	Set Nebulizer	0.3 Bar
Focus	Active	Set Capillary	4500 V	Set Dry Heater	200 °C
Scan Begin	50 m/z	Set End Plate Offset	-500 V	Set Dry Gas	4.0 l/min
Scan End	400 m/z	Set Charging Voltage	2000 V	Set Divert Valve	Source
		Set Corona	0 nA	Set APCI Heater	0 °C

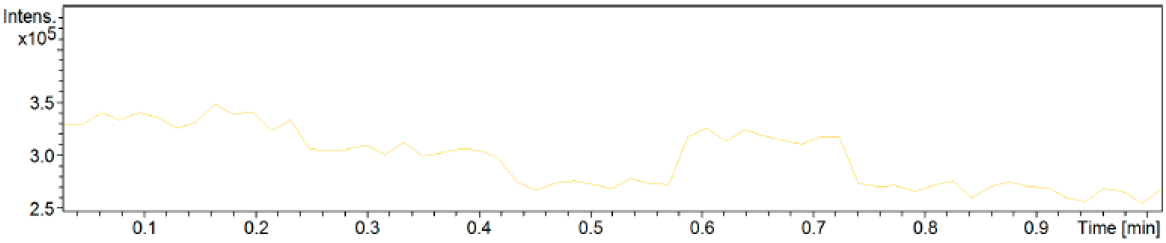

#	RT [min]	Area	Int. Type	I	S/N	Chromatogram	Max. m/z	FWHM [min]
n.a.	0.7	n.a.	Average spectrum	n.a.	n.a.	n.a.	344.0600	n.a.

+MS, 0.5-0.9min #26-53

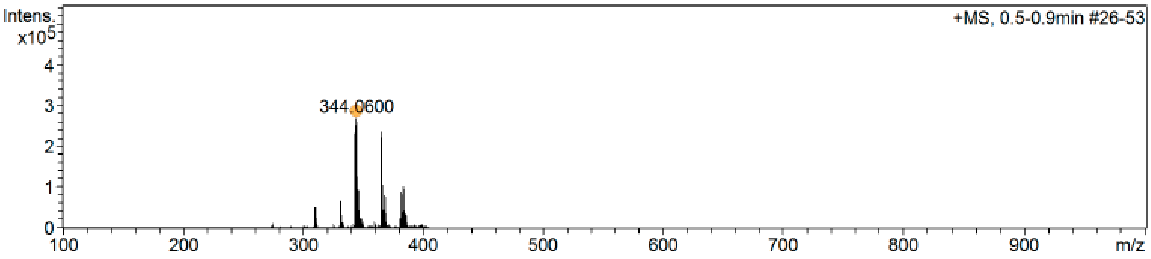

Meas. m/z	#	Ion Formula	m/z	err [ppm]	mSigma	# Sigma	Score	rdb	e⁻ Conf	N-Rule
344.0600	1	C17H12ClFN3O2	344.0597	-1.0	37.1	1	100.00	12.5	even	ok
	2	C22H12ClFN	344.0637	10.7	38.6	2	7.46	16.5	even	ok
	3	C13H8ClFN9	344.0570	8.8	42.0	3	13.06	13.5	even	ok
	4	C15H11ClF4N3	344.0572	-8.1	42.0	4	16.43	9.5	even	ok
	5	C14H13ClF2N3O3	344.0608	2.3	43.0	5	68.66	8.5	even	ok
	6	C14H13ClF6N	344.0635	-10.2	46.7	6	7.06	5.5	even	ok
	7	C14H16Cl2F4N	344.0590	-2.8	124.3	7	2.47	4.5	even	ok
	8	C13H18Cl2F2NO3	344.0626	7.6	126.9	8	0.65	3.5	even	ok
	9	C16H17Cl2FNO2	344.0615	-4.3	127.1	9	1.55	7.5	even	ok
	1	C20H13ClFNNa	344.0613	3.7	36.7	1	100.00	13.5	even	ok
	2	C17H14ClF2NNaO	344.0624	7.0	38.6	2	41.30	9.5	even	ok
	3	C15H13ClFN3NaO2	344.0573	-8.0	40.9	3	28.35	9.5	even	ok
	4	C14H15ClF3NNaO2	344.0636	-10.3	45.5	4	11.29	5.5	even	ok
	5	C14H18Cl2FNNaO2	344.0591	-2.7	126.1	5	3.68	4.5	even	ok

HRMS chat of compound **4e**

Acquisition Parameter

Source Type	ESI	Ion Polarity	Positive	Set Nebulizer	0.3 Bar
Focus	Active	Set Capillary	4500 V	Set Dry Heater	200 °C
Scan Begin	50 m/z	Set End Plate Offset	-500 V	Set Dry Gas	4.0 l/min
Scan End	400 m/z	Set Charging Voltage	2000 V	Set Divert Valve	Source
		Set Corona	0 nA	Set APCI Heater	0 °C

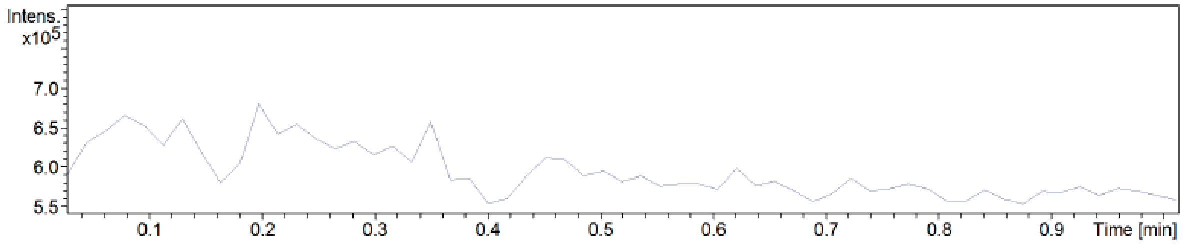

#	RT [min]	Area	Int. Type	I	S/N	Chromatogram	Max. m/z	FWHM [min]
n.a.	0.4	n.a.	Average spectrum	n.a.	n.a.	n.a.	343.0640	n.a.

+MS, 0.0-0.8min #2-45

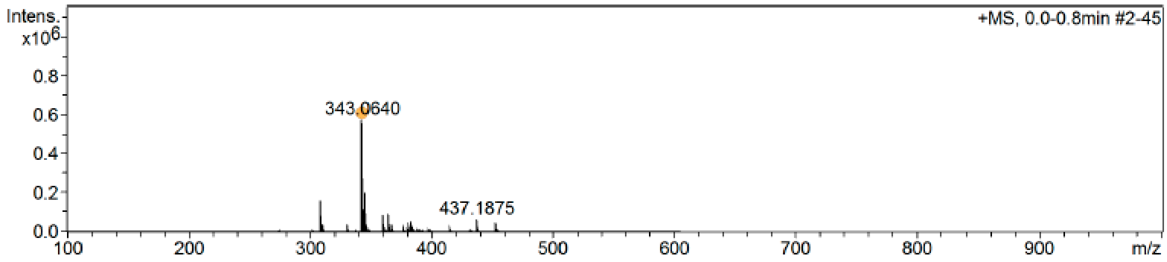

Meas. m/z	#	Ion Formula	m/z	err [ppm]	mSigma	# Sigma	Score	rdb	e⁻ Conf	N-Rule
343.0640	1	C18H13ClFN2O2	343.0644	-1.2	10.8	1	100.00	12.5	even	ok
	2	C14H9ClFN8	343.0617	6.7	17.9	2	26.02	13.5	even	ok
	3	C16H12ClF4N2	343.0620	-6.0	18.0	3	31.52	9.5	even	ok
	4	C15H14ClF2N2O3	343.0656	-4.5	20.0	4	43.56	8.5	even	ok
	5	C13H13ClFN4O4	343.0604	-10.6	25.6	5	6.09	8.5	even	ok
	6	C13H13ClF5N2O	343.0631	2.6	30.5	6	50.48	5.5	even	ok
	7	C15H17Cl2F4	343.0638	0.6	128.9	7	1.56	4.5	even	ok
	8	C13H14Cl2FN6	343.0636	-1.3	129.0	8	1.39	8.5	even	ok
	9	C14H19Cl2F2O3	343.0674	9.8	131.4	9	0.14	3.5	even	ok
	10	C17H18Cl2FO2	343.0662	-6.5	132.6	10	0.37	7.5	even	ok
	1	C18H15ClF2NaO	343.0672	9.2	12.7	1	24.63	9.5	even	ok
	2	C21H14ClFNa	343.0660	-5.9	14.9	2	64.80	13.5	even	ok
	3	C16H14ClFN2NaO2	343.0620	-5.9	16.4	3	63.07	9.5	even	ok
	4	C13H15ClF2N2NaO3	343.0631	2.5	29.3	4	100.00	5.5	even	ok
	5	C13H15ClF6Na	343.0659	5.4	35.8	5	36.10	2.5	even	ok
	6	C13H18Cl2F4Na	343.0614	-7.6	128.4	6	0.63	1.5	even	ok
	7	C15H19Cl2FNaO2	343.0638	0.5	130.9	7	2.66	4.5	even	ok

HRMS chat of compound **4g**

Acquisition Parameter

Source Type	ESI	Ion Polarity	Positive	Set Nebulizer	0.3 Bar
Focus	Active	Set Capillary	4500 V	Set Dry Heater	200 °C
Scan Begin	50 m/z	Set End Plate Offset	-500 V	Set Dry Gas	4.0 l/min
Scan End	420 m/z	Set Charging Voltage	2000 V	Set Divert Valve	Source
		Set Corona	0 nA	Set APCI Heater	0 °C

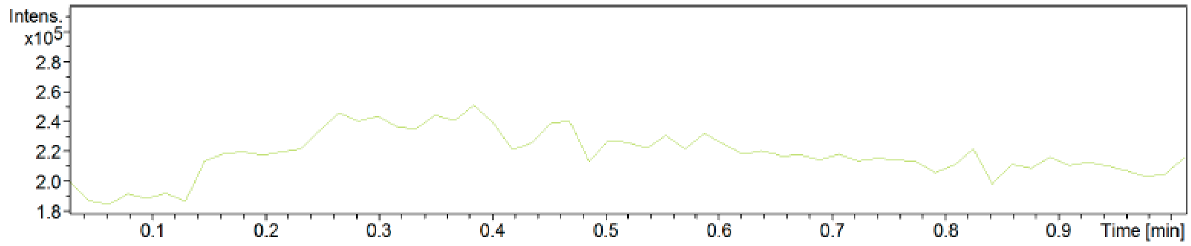

#	RT [min]	Area	Int. Type	I	S/N	Chromatogram	Max. m/z	FWHM [min]
n.a.	0.5	n.a.	Average spectrum	n.a.	n.a.	n.a.	344.0601	n.a.

+MS, 0.1-0.9min #6-55

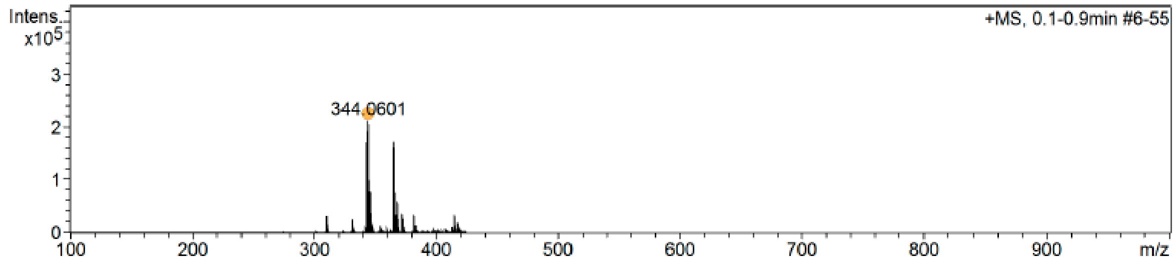

Meas. m/z	#	Ion Formula	m/z	err [ppm]	mSigma	# Sigma	Score	rdb	e⁻ Conf	N-Rule
344.0601	1	C17H12ClFN3O2	344.0597	-1.2	19.8	1	100.00	12.5	even	ok
	2	C22H12ClFN	344.0637	10.5	20.6	2	8.17	16.5	even	ok
	3	C13H8ClFN9	344.0570	-9.0	28.7	3	11.97	13.5	even	ok
	4	C15H11ClF4N3	344.0572	8.3	28.7	4	15.08	9.5	even	ok
	5	C14H13ClF2N3O3	344.0608	-2.2	30.4	5	66.13	8.5	even	ok
	6	C14H13ClF6N	344.0635	-10.1	35.6	6	6.79	5.5	even	ok
	7	C14H16Cl2F4N	344.0590	2.9	122.0	7	1.86	4.5	even	ok
	8	C13H18Cl2F2NO3	344.0626	-7.5	124.7	8	0.51	3.5	even	ok
	9	C16H17Cl2FNO2	344.0615	-4.2	124.9	9	1.22	7.5	even	ok
	1	C20H13ClFNNa	344.0613	3.5	17.9	1	100.00	13.5	even	ok
	2	C17H14ClF2NNaO	344.0624	-6.9	22.8	2	39.47	9.5	even	ok
	3	C15H13ClFN3NaO2	344.0573	8.1	26.9	3	24.32	9.5	even	ok
	4	C14H15ClF3NNaO2	344.0636	-10.2	34.1	4	10.12	5.5	even	ok
	5	C14H18Cl2FNNaO2	344.0591	2.8	123.9	5	2.55	4.5	even	ok

HRMS chat of compound **4i**

Acquisition Parameter

Source Type	ESI	Ion Polarity	Positive	Set Nebulizer	0.3 Bar
Focus	Active	Set Capillary	4500 V	Set Dry Heater	200 °C
Scan Begin	50 m/z	Set End Plate Offset	-500 V	Set Dry Gas	4.0 l/min
Scan End	600 m/z	Set Charging Voltage	2000 V	Set Divert Valve	Source
		Set Corona	0 nA	Set APCI Heater	0 °C

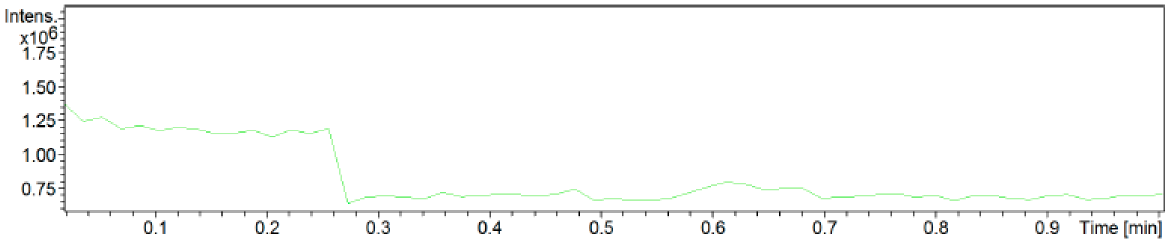

#	RT [min]	Area	Int. Type	I	S/N	Chromatogram	Max. m/z	FWHM [min]
n.a.	0.6	n.a.	Average spectrum	n.a.	n.a.	n.a.	366.0415	n.a.

+MS, 0.2-1.0min #9-57

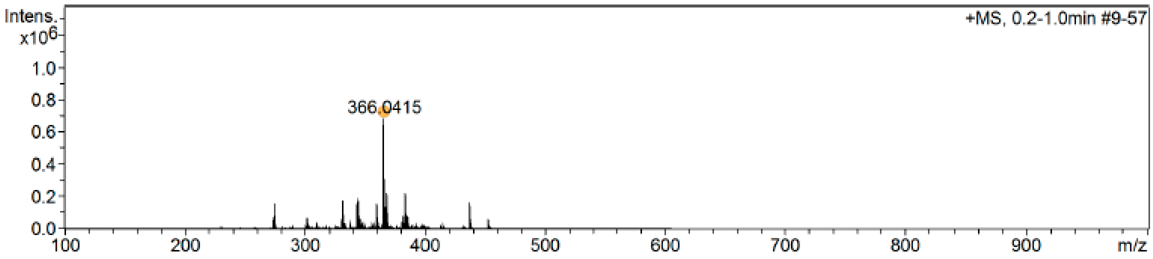

Meas. m/z	#	Ion Formula	m/z	err [ppm]	mSigma	# Sigma	Score	rdb	e⁻ Conf	N-Rule
366.0415	1	C17H11ClFN3NaO2	366.0416	0.2	4.9	1	100.00	12.5	even	ok
	2	C15H10ClF4N3Na	366.0392	-6.4	11.9	2	21.86	9.5	even	ok
	3	C13H7ClFN9Na	366.0389	-7.1	11.9	3	17.77	13.5	even	ok
	4	C14H12ClF2N3NaO3	366.0427	3.4	16.3	4	45.02	8.5	even	ok
	5	C14H12ClF6NNa	366.0455	-10.8	20.2	5	3.71	5.5	even	ok
	6	C12H9ClF3N7Na	366.0452	10.1	20.2	6	4.89	9.5	even	ok
	7	C12H11ClFN5NaO4	366.0376	-10.7	22.8	7	3.60	8.5	even	ok
	8	C12H11ClF5N3NaO	366.0403	-3.3	26.7	8	36.32	5.5	even	ok
	9	C14H15Cl2F4NNa	366.0410	1.4	138.7	9	0.49	4.5	even	ok
	10	C12H12Cl2FN7Na	366.0407	-2.1	138.8	10	0.44	8.5	even	ok
	11	C13H17Cl2F2NNaO3	366.0446	-8.4	141.3	11	0.07	3.5	even	ok
	12	C16H16Cl2FNNaO2	366.0434	5.2	142.4	12	0.17	7.5	even	ok

HRMS chat of compound **4j**

Acquisition Parameter

Source Type	ESI	Ion Polarity	Positive	Set Nebulizer	0.3 Bar
Focus	Active	Set Capillary	4500 V	Set Dry Heater	200 °C
Scan Begin	50 m/z	Set End Plate Offset	-500 V	Set Dry Gas	4.0 l/min
Scan End	600 m/z	Set Charging Voltage	2000 V	Set Divert Valve	Source
		Set Corona	0 nA	Set APCI Heater	0 °C

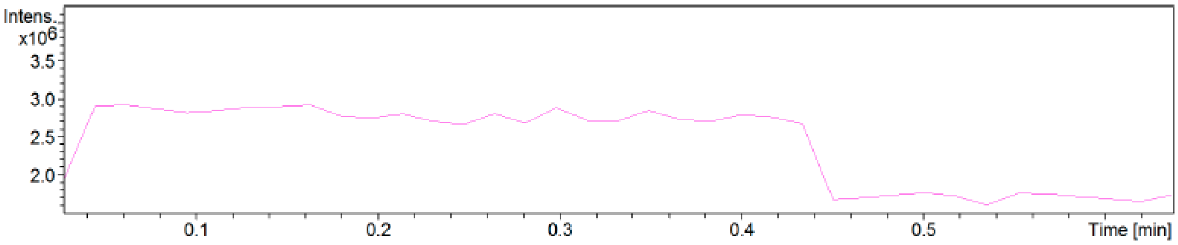

#	RT [min]	Area	Int. Type	I	S/N	Chromatogram	Max. m/z	FWHM [min]
n.a.	0.1	n.a.	Average spectrum	n.a.	n.a.	n.a.	343.0648	n.a.

+MS, 0.0-0.1min #2-5

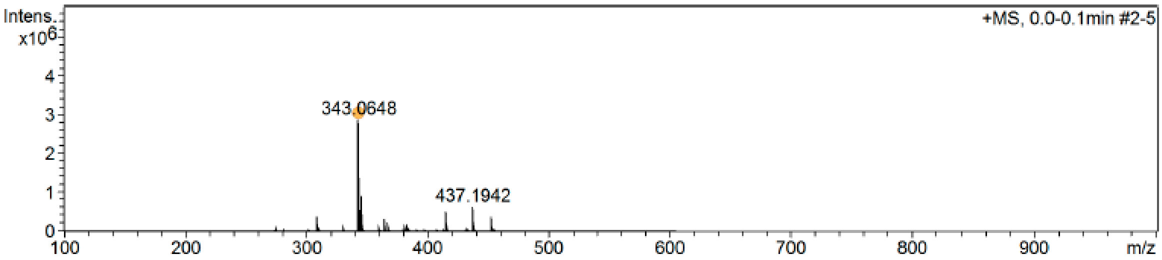

Meas. m/z	#	Ion Formula	m/z	err [ppm]	mSigma	# Sigma	Score	rdb	e⁻ Conf	N-Rule
343.0648	1	C16H12ClF4N2	343.0620	-8.2	9.7	1	20.58	9.5	even	ok
	2	C14H9ClFN8	343.0617	8.9	9.7	2	16.33	13.5	even	ok
	3	C15H14ClF2N2O3	343.0656	-2.2	12.8	3	86.95	8.5	even	ok
	4	C15H14ClF6	343.0683	10.1	13.6	4	9.93	5.5	even	ok
	5	C13H11ClF3N6	343.0680	9.4	13.7	5	12.77	9.5	even	ok
	6	C18H13ClFN2O2	343.0644	-1.1	15.1	6	100.00	12.5	even	ok
	7	C13H13ClF5N2O	343.0631	4.9	19.2	7	43.28	5.5	even	ok
	8	C11H10ClF2N8O	343.0629	-5.6	19.2	8	36.34	9.5	even	ok
	9	C12H15ClF3N2O4	343.0667	5.5	23.7	9	33.53	4.5	even	ok
	10	C23H13ClF	343.0684	-10.6	33.7	10	5.33	16.5	even	ok
	11	C12H18Cl2F5O	343.0649	-0.4	143.2	11	0.73	0.5	even	ok
	12	C15H17Cl2F4	343.0638	2.9	143.8	12	0.46	4.5	even	ok
	13	C13H14Cl2FN6	343.0636	-3.6	143.9	13	0.40	8.5	even	ok
	14	C11H20Cl2F3O4	343.0685	-10.9	146.0	14	0.04	-0.5	even	ok
	15	C14H19Cl2F2O3	343.0674	7.5	146.0	15	0.13	3.5	even	ok
	16	C12H18Cl2FN2O4	343.0622	-7.5	146.3	16	0.13	3.5	even	ok
	17	C17H18Cl2FO2	343.0662	4.2	148.2	17	0.26	7.5	even	ok
	18	C12H19Cl3FN4	343.0654	-1.7	256.8	18	0.00	3.5	even	ok
	1	C16H14ClFN2NaO2	343.0620	-8.1	11.0	1	39.38	9.5	even	ok

HRMS chat of compound **4k**

Acquisition Parameter

Source Type	ESI	Ion Polarity	Positive	Set Nebulizer	0.3 Bar
Focus	Active	Set Capillary	4500 V	Set Dry Heater	200 °C
Scan Begin	50 m/z	Set End Plate Offset	-500 V	Set Dry Gas	4.0 l/min
Scan End	600 m/z	Set Charging Voltage	2000 V	Set Divert Valve	Source
		Set Corona	0 nA	Set APCI Heater	0 °C

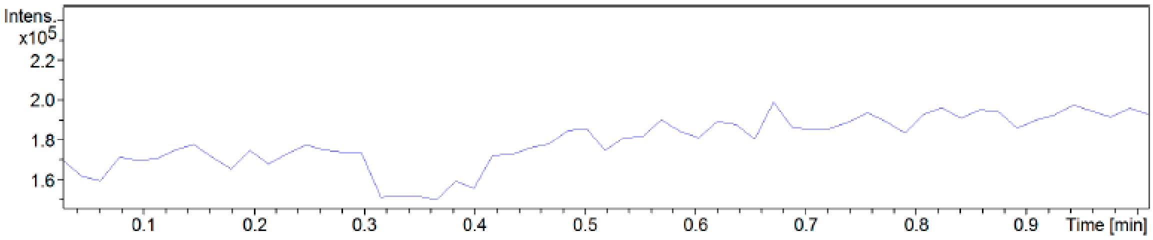

#	RT [min]	Area	Int. Type	I	S/N	Chromatogram	Max. m/z	FWHM [min]
n.a.	0.6	n.a.	Average spectrum	n.a.	n.a.	n.a.	343.0639	n.a.

+MS, 0.3-1.0min #16-57

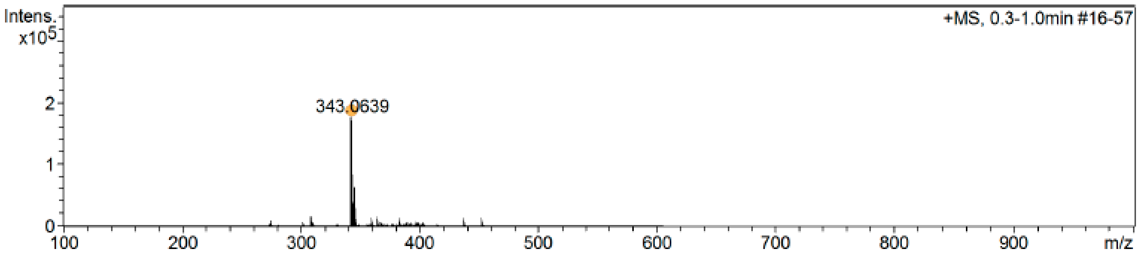

Meas. m/z	#	Ion Formula	m/z	err [ppm]	mSigma	# Sigma	Score	rdb	e⁻ Conf	N-Rule
343.0639	1	C18H13ClFN2O2	343.0644	1.6	8.2	1	100.00	12.5	even	ok
	2	C16H12ClF4N2	343.0620	5.5	18.6	2	35.85	9.5	even	ok
	3	C14H9ClFN8	343.0617	6.2	18.6	3	29.82	13.5	even	ok
	4	C15H14ClF2N2O3	343.0656	-4.9	21.2	4	39.11	8.5	even	ok
	5	C13H13ClFN4O4	343.0604	-10.1	27.4	5	7.08	8.5	even	ok
	6	C13H13ClF5N2O	343.0631	-2.2	32.5	6	53.58	5.5	even	ok
	7	C15H17Cl2F4	343.0638	-0.2	128.9	7	1.70	4.5	even	ok
	8	C13H14Cl2FN6	343.0636	-0.9	129.0	8	1.53	8.5	even	ok
	9	C14H19Cl2F2O3	343.0674	10.3	131.6	9	0.12	3.5	even	ok
	10	C17H18Cl2FO2	343.0662	6.9	132.4	10	0.33	7.5	even	ok
	1	C21H14ClFNa	343.0660	-6.3	9.7	1	61.38	13.5	even	ok
	2	C18H15ClF2NaO	343.0672	9.7	11.7	2	20.75	9.5	even	ok
	3	C16H14ClFN2NaO2	343.0620	5.4	17.0	3	67.66	9.5	even	ok
	4	C13H15ClF2N2NaO3	343.0631	-2.1	31.3	4	100.00	5.5	even	ok
	5	C13H15ClF6Na	343.0659	5.9	40.4	5	27.53	2.5	even	ok
	6	C13H18Cl2F4Na	343.0614	-7.2	128.6	6	0.68	1.5	even	ok
	7	C15H19Cl2FNaO2	343.0638	0.1	130.9	7	2.73	4.5	even	ok

HRMS chat of compound **4l**

Acquisition Parameter

Source Type	ESI	Ion Polarity	Positive	Set Nebulizer	0.3 Bar
Focus	Active	Set Capillary	4500 V	Set Dry Heater	200 °C
Scan Begin	50 m/z	Set End Plate Offset	-500 V	Set Dry Gas	4.0 l/min
Scan End	400 m/z	Set Charging Voltage	2000 V	Set Divert Valve	Source
		Set Corona	0 nA	Set APCI Heater	0 °C

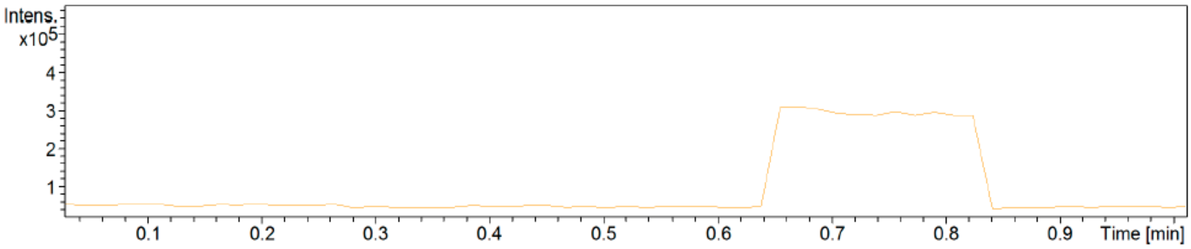

#	RT [min]	Area	Int. Type	I	S/N	Chromatogram	Max. m/z	FWHM [min]
n.a.	0.5	n.a.	Average spectrum	n.a.	n.a.	n.a.	344.0596	n.a.

+MS, 0.3-0.7min #17-38

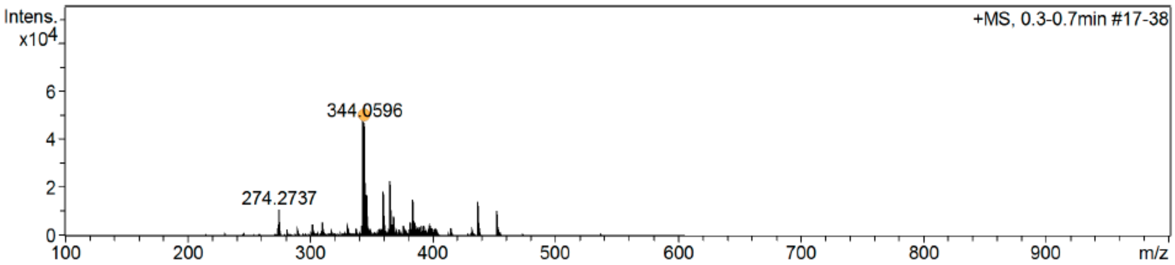

Meas. m/z	#	Ion Formula	m/z	err [ppm]	mSigma	# Sigma	Score	rdb	e⁻ Conf	N-Rule
344.0596	1	C17H12ClFN3O2	344.0597	0.0	24.3	1	100.00	12.5	even	ok
	2	C15H11ClF4N3	344.0572	7.1	34.6	2	17.53	9.5	even	ok
	3	C16H17Cl2FNO2	344.0615	5.4	127.9	3	0.72	7.5	even	ok
	1	C20H13ClFNNa	344.0613	-4.7	17.6	1	100.00	13.5	even	ok
	2	C17H14ClF2NNaO	344.0624	8.1	27.8	2	31.80	9.5	even	ok
	3	C15H13ClFN3NaO2	344.0573	-7.0	33.2	3	38.93	9.5	even	ok

HRMS chat of compound **4m**

Acquisition Parameter

Source Type	ESI	Ion Polarity	Positive	Set Nebulizer	0.3 Bar
Focus	Active	Set Capillary	4500 V	Set Dry Heater	200 °C
Scan Begin	50 m/z	Set End Plate Offset	-500 V	Set Dry Gas	4.0 l/min
Scan End	600 m/z	Set Charging Voltage	2000 V	Set Divert Valve	Source
		Set Corona	0 nA	Set APCI Heater	0 °C

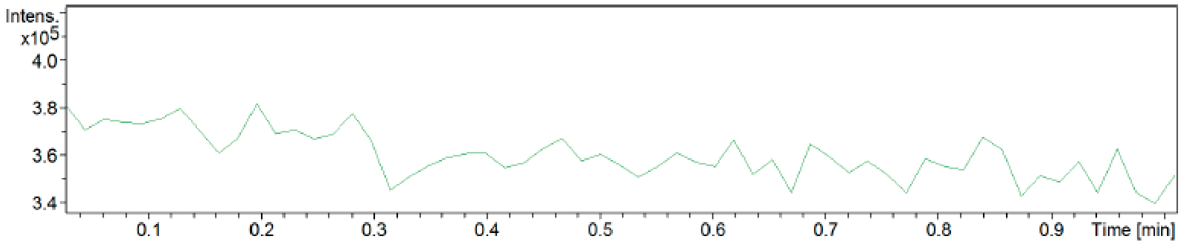

#	RT [min]	Area	Int. Type	I	S/N	Chromatogram	Max. m/z	FWHM [min]
n.a.	0.4	n.a.	Average spectrum	n.a.	n.a.	n.a.	359.0346	n.a.

+MS, 0.1-0.7min #3-39

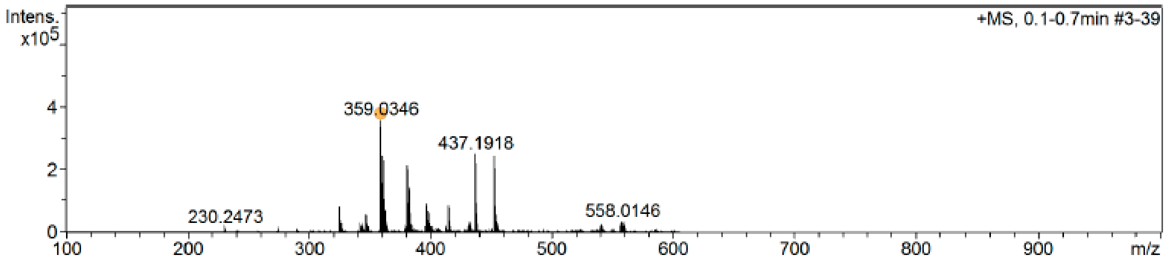

Meas. m/z	#	Ion Formula	m/z	err [ppm]	mSigma	# Sigma	Score	rdb	e⁻ Conf	N-Rule
359.0346	1	C18H13Cl2N2O2	359.0349	-0.6	36.1	1	100.00	12.5	even	ok
	2	C14H9Cl2N8	359.0322	-6.9	42.1	2	20.19	13.5	even	ok
	3	C13H13Cl2N4O4	359.0308	10.6	46.4	3	4.69	8.5	even	ok
	4	C13H14Cl3N6	359.0340	-1.8	113.2	4	5.49	8.5	even	ok
	5	C17H18Cl3O2	359.0367	-5.7	116.6	5	1.88	7.5	even	ok
	6	C24H8ClN2	359.0371	6.7	172.1	6	0.03	21.5	even	ok
	7	C18H12ClO6	359.0317	8.2	173.5	7	0.01	12.5	even	ok
	8	C19H8ClN4O2	359.0330	-4.5	174.5	8	0.04	17.5	even	ok
	1	C21H14Cl2Na	359.0365	5.1	36.0	1	100.00	13.5	even	ok
	2	C16H14Cl2N2NaO2	359.0325	6.1	40.4	2	67.62	9.5	even	ok
	3	C15H19Cl3NaO2	359.0343	1.0	115.0	3	14.43	4.5	even	ok
	4	C22H9ClN2Na	359.0346	-0.0	173.9	4	0.25	18.5	even	ok

HRMS chat of compound **4n**

Acquisition Parameter

Source Type	ESI	Ion Polarity	Positive	Set Nebulizer	0.3 Bar
Focus	Active	Set Capillary	4500 V	Set Dry Heater	200 °C
Scan Begin	50 m/z	Set End Plate Offset	-500 V	Set Dry Gas	4.0 l/min
Scan End	600 m/z	Set Charging Voltage	2000 V	Set Divert Valve	Source
		Set Corona	0 nA	Set APCI Heater	0 °C

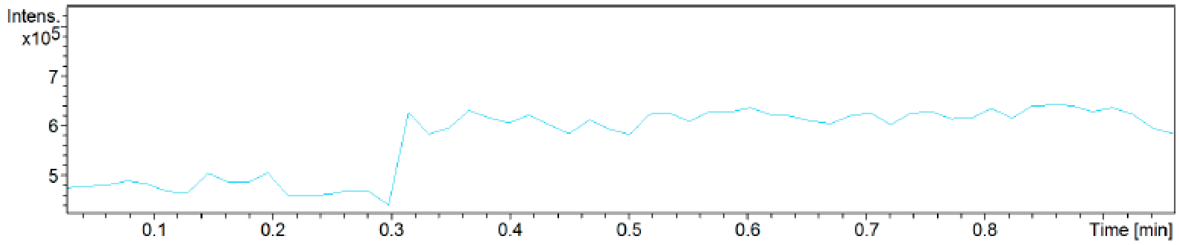

#	RT [min]	Area	Int. Type	I	S/N	Chromatogram	Max. m/z	FWHM [min]
n.a.	0.3	n.a.	Average spectrum	n.a.	n.a.	n.a.	382.0123	n.a.

+MS, 0.0-0.5min #2-31

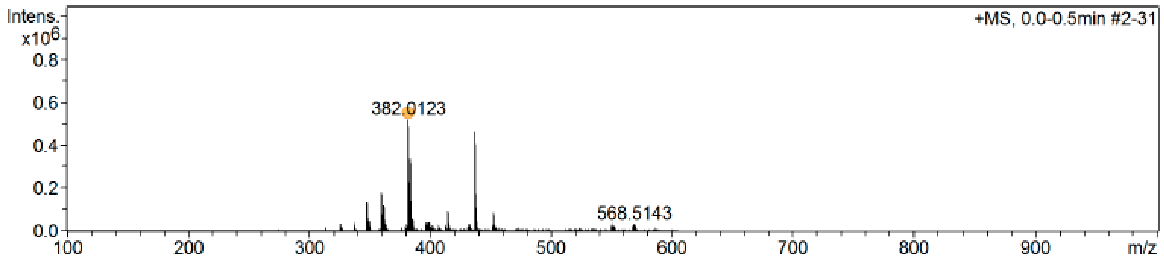

Meas. m/z	#	Ion Formula	m/z	err [ppm]	mSigma	# Sigma	Score	rdb	e⁻ Conf	N-Rule
382.0123	1	C15H6Cl2N9	382.0118	1.3	6.3	1	100.00	16.5	even	ok
	2	C19H10Cl2N3O2	382.0145	5.7	9.8	2	32.45	15.5	even	ok
	3	C14H10Cl2N5O4	382.0104	-4.8	17.0	3	36.35	11.5	even	ok
	4	C13H14Cl2NO8	382.0091	8.3	28.3	4	8.86	6.5	even	ok
	5	C14H11Cl3N7	382.0136	-3.5	138.2	5	0.54	11.5	even	ok
	6	C13H15Cl3N3O4	382.0123	-0.0	140.4	6	0.92	6.5	even	ok
	7	C19H9ClNO6	382.0113	2.6	142.8	7	0.31	15.5	even	ok
	8	C20H5ClN5O2	382.0126	-0.9	144.3	8	0.39	20.5	even	ok
	9	C15H5ClN7O4	382.0086	-9.6	147.7	9	0.02	16.5	even	ok
	10	C16HClN11	382.0099	-6.1	148.6	10	0.08	21.5	even	ok
	11	C13H16Cl4N5	382.0154	8.3	192.4	11	0.00	6.5	even	ok
	1	C17H11Cl2N3NaO2	382.0121	0.6	7.0	1	100.00	12.5	even	ok
	2	C13H7Cl2N9Na	382.0094	-7.6	15.8	2	13.51	13.5	even	ok
	3	C22H11Cl2NNa	382.0161	9.9	19.9	3	4.94	16.5	even	ok
	4	C16H16Cl3NNaO2	382.0139	4.2	140.3	4	0.35	7.5	even	ok
	5	C23H6ClN3Na	382.0142	5.1	144.3	5	0.13	21.5	even	ok
	6	C17H10ClNNaO6	382.0089	8.9	145.6	6	0.03	12.5	even	ok
	7	C18H6ClN5NaO2	382.0102	5.4	146.5	7	0.11	17.5	even	ok

HRMS chat of compound **4o**

Acquisition Parameter

Source Type	ESI	Ion Polarity	Positive	Set Nebulizer	0.3 Bar
Focus	Active	Set Capillary	4500 V	Set Dry Heater	200 °C
Scan Begin	50 m/z	Set End Plate Offset	-500 V	Set Dry Gas	4.0 l/min
Scan End	600 m/z	Set Charging Voltage	2000 V	Set Divert Valve	Source
		Set Corona	0 nA	Set APCI Heater	0 °C

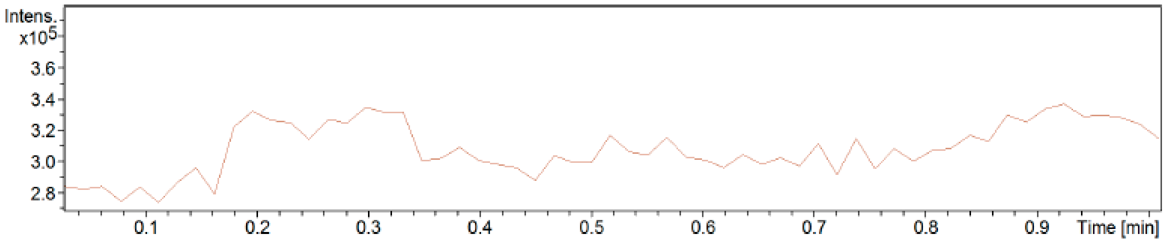

#	RT [min]	Area	Int. Type	I	S/N	Chromatogram	Max. m/z	FWHM [min]
n.a.	0.2	n.a.	Average spectrum	n.a.	n.a.	n.a.	360.0304	n.a.

+MS, 0.1-0.3min #3-19

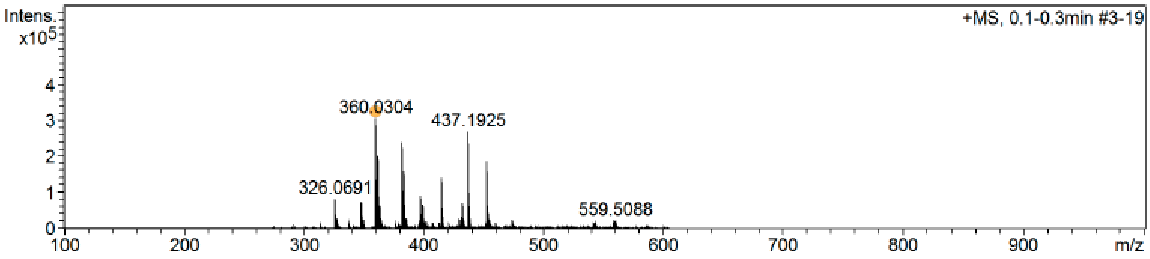

Meas. m/z	#	Ion Formula	m/z	err [ppm]	mSigma	# Sigma	Score	rdb	e^- Conf	N-Rule
360.0304	1	C17H12Cl2N3O2	360.0301	-0.8	40.1	1	100.00	12.5	even	ok
	2	C22H12Cl2N	360.0341	-10.4	43.1	2	6.43	16.5	even	ok
	3	C13H8Cl2N9	360.0274	8.3	44.6	3	13.63	13.5	even	ok
	4	C12H13Cl3N7	360.0293	-3.2	117.6	4	3.77	8.5	even	ok
	5	C16H17Cl3NO2	360.0319	-4.3	121.0	5	2.44	7.5	even	ok
	6	C23H7ClN3	360.0323	-5.3	168.0	6	0.06	21.5	even	ok
	7	C17H11ClNO6	360.0269	-9.6	168.6	7	0.01	12.5	even	ok
	8	C18H7ClN5O2	360.0283	-5.9	169.8	8	0.04	17.5	even	ok
	1	C20H13Cl2NNa	360.0317	3.7	40.7	1	100.00	13.5	even	ok
	2	C15H13Cl2N3NaO2	360.0277	-7.5	43.2	2	32.70	9.5	even	ok
	3	C14H18Cl3NNaO2	360.0295	2.4	119.5	3	7.11	4.5	even	ok
	4	C21H8ClN3Na	360.0299	1.4	169.5	4	0.22	18.5	even	ok

HRMS chat of compound **4p**

Acquisition Parameter

Source Type	ESI	Ion Polarity	Positive	Set Nebulizer	0.3 Bar
Focus	Active	Set Capillary	4500 V	Set Dry Heater	200 °C
Scan Begin	50 m/z	Set End Plate Offset	-500 V	Set Dry Gas	4.0 l/min
Scan End	600 m/z	Set Charging Voltage	2000 V	Set Divert Valve	Source
		Set Corona	0 nA	Set APCI Heater	0 °C

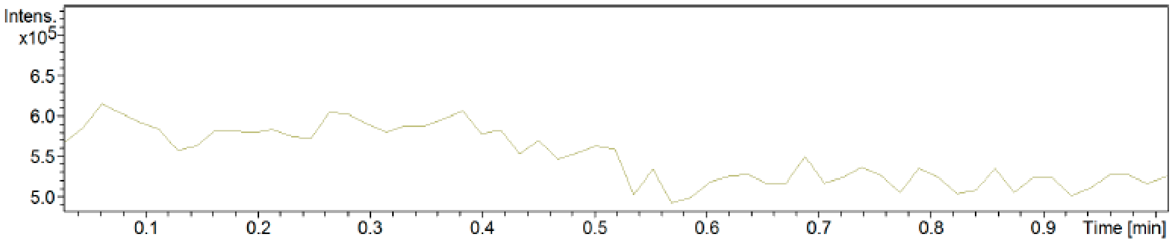

#	RT [min]	Area	Int. Type	I	S/N	Chromatogram	Max. m/z	FWHM [min]
n.a.	0.8	n.a.	Average spectrum	n.a.	n.a.	n.a.	359.0351	n.a.

+MS, 0.6-1.0min #36-57

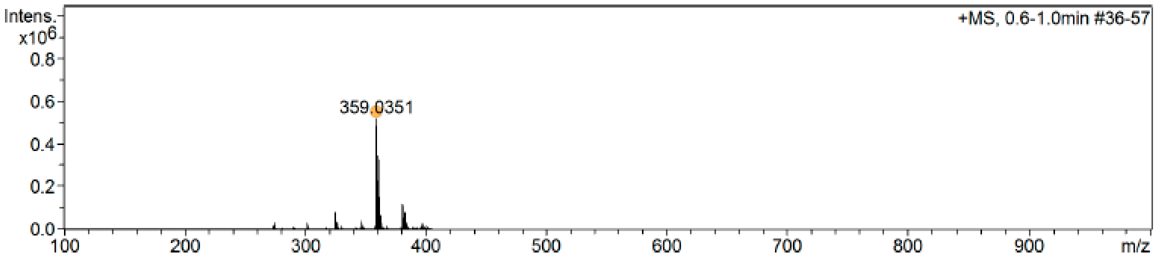

Meas. m/z	#	Ion Formula	m/z	err [ppm]	mSigma	# Sigma	Score	rdb	e⁻ Conf	N-Rule
359.0351	1	C18H13Cl2N2O2	359.0349	0.7	7.3	1	100.00	12.5	even	ok
	2	C14H9Cl2N8	359.0322	8.1	18.1	2	12.90	13.5	even	ok
	3	C23H13Cl2	359.0389	-10.5	18.7	3	5.16	16.5	even	ok
	4	C13H14Cl3N6	359.0340	3.0	128.8	4	0.97	8.5	even	ok
	5	C17H18Cl3O2	359.0367	4.4	132.7	5	0.56	7.5	even	ok
	6	C24H8ClN2	359.0371	-5.4	154.1	6	0.07	21.5	even	ok
	7	C18H12ClO6	359.0317	-9.5	155.4	7	0.02	12.5	even	ok
	8	C19H8ClN4O2	359.0330	5.8	156.4	8	0.06	17.5	even	ok
	1	C21H14Cl2Na	359.0365	-3.8	9.4	1	100.00	13.5	even	ok
	2	C16H14Cl2N2NaO2	359.0325	7.4	16.6	2	33.08	9.5	even	ok
	3	C15H19Cl3NaO2	359.0343	2.3	130.9	3	1.94	4.5	even	ok
	4	C22H9ClN2Na	359.0346	1.3	155.8	4	0.30	18.5	even	ok

HRMS chat of compound **4r**

Acquisition Parameter

Source Type	ESI	Ion Polarity	Positive	Set Nebulizer	0.3 Bar
Focus	Active	Set Capillary	4500 V	Set Dry Heater	200 °C
Scan Begin	50 m/z	Set End Plate Offset	-500 V	Set Dry Gas	4.0 l/min
Scan End	600 m/z	Set Charging Voltage	2000 V	Set Divert Valve	Source
		Set Corona	0 nA	Set APCI Heater	0 °C

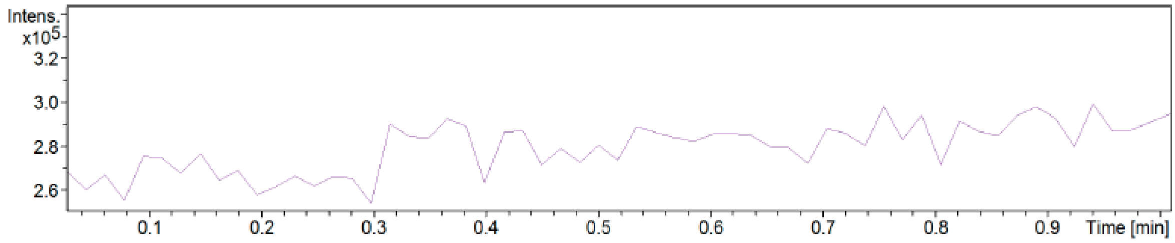

#	RT [min]	Area	Int. Type	I	S/N	Chromatogram	Max. m/z	FWHM [min]
n.a.	0.5	n.a.	Average spectrum	n.a.	n.a.	n.a.	360.0304	n.a.

+MS, 0.2-0.8min #10-49

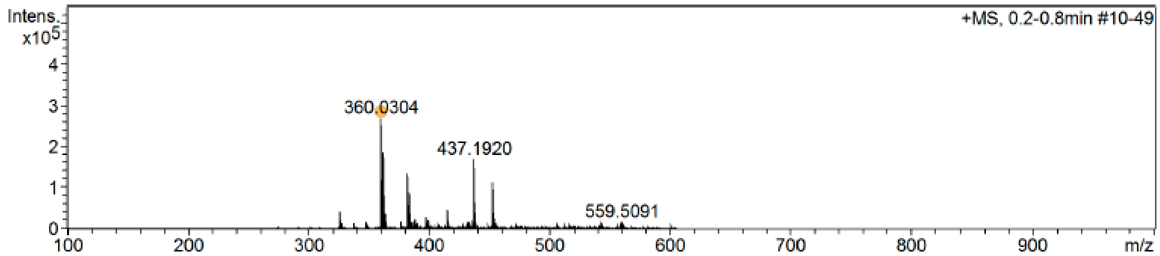

Meas. m/z	#	Ion Formula	m/z	err [ppm]	mSigma	# Sigma	Score	rdb	e⁻ Conf	N-Rule
360.0304	1	C17H12Cl2N3O2	360.0301	0.9	16.9	1	100.00	12.5	even	ok
	2	C22H12Cl2N	360.0341	10.3	21.2	2	6.79	16.5	even	ok
	3	C13H8Cl2N9	360.0274	-8.4	26.1	3	12.41	13.5	even	ok
	4	C12H13Cl3N7	360.0293	3.3	119.3	4	1.97	8.5	even	ok
	5	C16H17Cl3NO2	360.0319	4.2	122.9	5	1.32	7.5	even	ok
	6	C23H7ClN3	360.0323	-5.2	165.5	6	0.04	21.5	even	ok
	7	C17H11ClNO6	360.0269	9.7	166.7	7	0.01	12.5	even	ok
	8	C18H7ClN5O2	360.0283	-6.0	167.8	8	0.03	17.5	even	ok
	1	C20H13Cl2NNa	360.0317	-3.6	16.7	1	100.00	13.5	even	ok
	2	C15H13Cl2N3NaO2	360.0277	-7.6	24.2	2	28.14	9.5	even	ok
	3	C14H18Cl3NNaO2	360.0295	2.5	121.3	3	3.45	4.5	even	ok
	4	C21H8ClN3Na	360.0299	1.5	167.3	4	0.14	18.5	even	ok
